# Supplementary figures and images for: The R203M and D377Y mutations of the nucleocapsid protein promote SARS-CoV-2 infectivity by impairing RIG-I-mediated antiviral signaling
Source: PLoS Pathog. 2025 Jan 22;21(1):e1012886. doi: 10.1371/journal.ppat.1012886 (PMC11771877; doi:10.1371/journal.ppat.1012886)

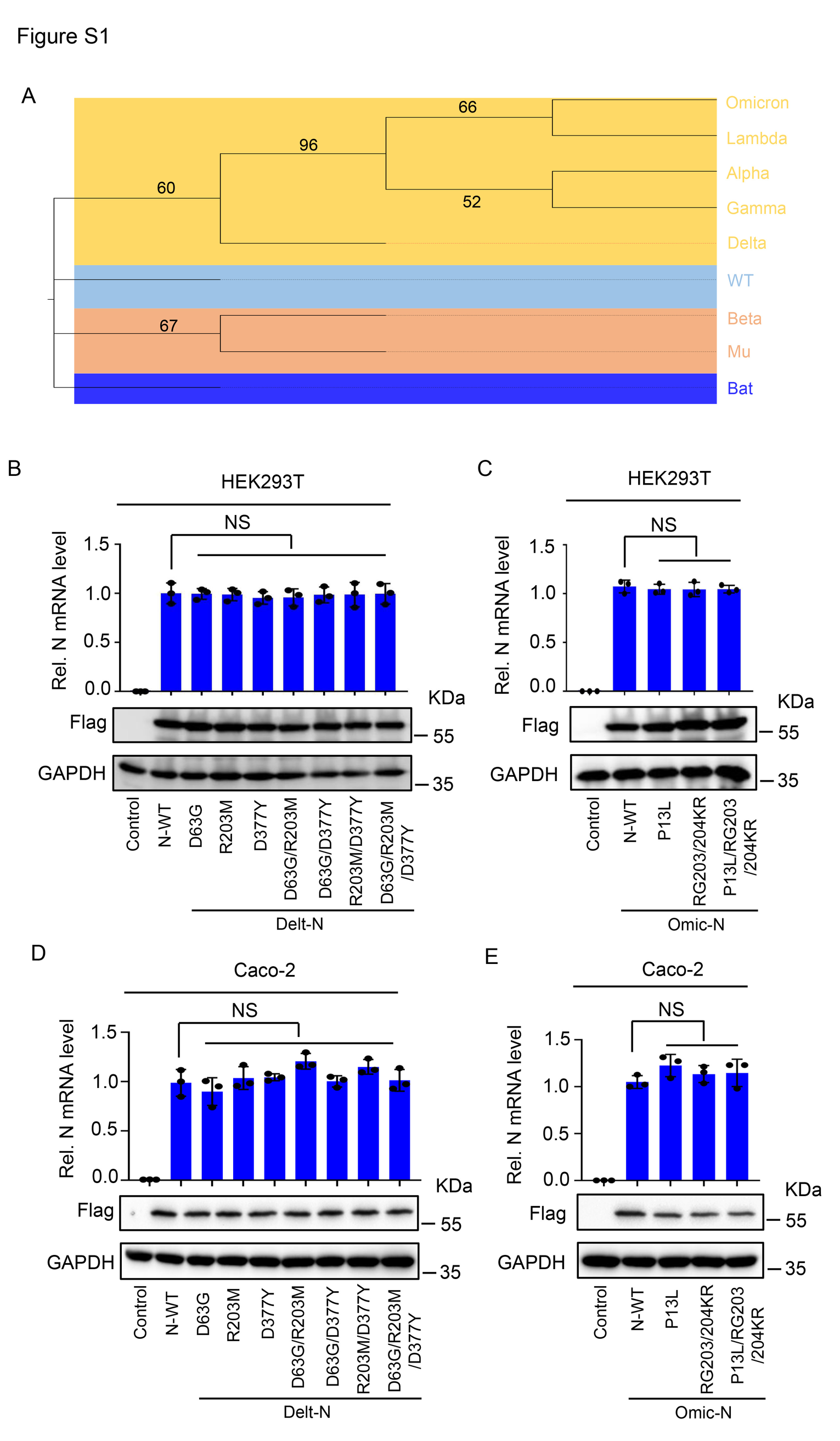

Supplement: S1 Fig — (A) A phylogenetic tree constructed with N protein of different variants, with Bootstrap values greater than 50 is labeled on the branches. (B–E) HEK293T or Caco-2 cells were transfected with plasmids encoding Delta (B, D) or Omicron (C, E) mutated N protein for 24 h. The total RNA in the cells was extracted and the mRNA level of N protein was detected by RT-PCR. Cell lysates were analyzed by immunoblotting. Data are representative of three independent experiments and one representative is shown (B–E). Error bars indicate SD of technical triplicates. Values are mean ± SEM. *P ≤ 0.05, **P ≤ 0.01, ***P ≤ 0.001, NS means No significant difference, two-tailed Student’s t-test. (TIF) [file ppat.1012886.s001.tif]

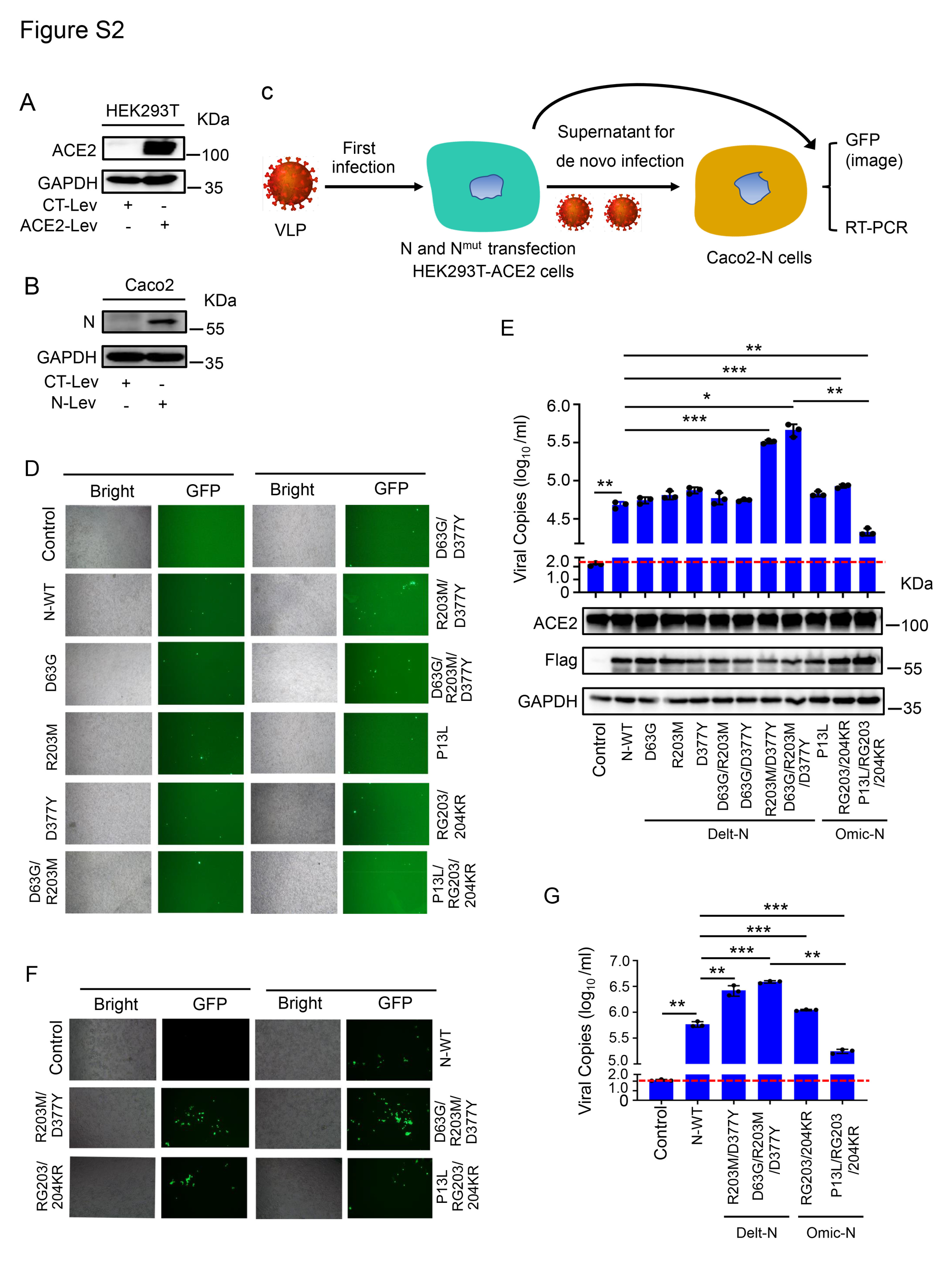

Supplement: S2 Fig — (A) HEK293T cells were stably infected with Lentivirus-CT or Lentivirus-ACE2, Cell lysates were analyzed by immunoblotting. (B) Caco-2 cells were stably infected with Lentivirus-CT or Lentivirus-N, Cell lysates were analyzed by immunoblotting. (C) Experimental scheme. HEK293T stable expression of ACE2 (HEK293T-ACE2) cells were transfected with plasmids encoding different mutated N protein for 24 h, and then infected with SARS-CoV-2 GFP/ΔN (MOI = 5) for 2 h, washed and incubated for an additional 48 h. The cell culture medium was collected to infect the Caco-2 stable expression of N (Caco-2-N) cells for 48 h. GFP fluorescence was observed by microscopy and viral RNA in supernatant was determined by RT-qPCR assay. (D) GFP expression was observed in HEK293T-ACE2 cells using microscopy at indicated time after inoculation. (E) The total RNA in supernatant of HEK293T-ACE2 cells was extracted and RT-qPCR assays were conducted to determine viral copies. Cell lysates were analyzed by immunoblotting. (F) GFP expression was observed in Caco-2-N cells using microscopy at indicated time after inoculation. (G) The total RNA in supernatant of Caco-2-N cells was extracted and RT-qPCR assays were conducted to determine viral copies. CT-Lev means CT- Lentivirus (A, B). ACE2-Lev means ACE2- Lentivirus (A). N-Lev means N- Lentivirus (B). Control means pcDNA3.1(+)-3 × flag empty plasmid (D–G). Data are representative of three independent experiments and one representative is shown. Error bars indicate SD of technical triplicates. Values are mean ± SEM. *P ≤ 0.05, **P ≤ 0.01, ***P ≤ 0.001, two-tailed Student’s t-test. (TIF) [file ppat.1012886.s002.tif]

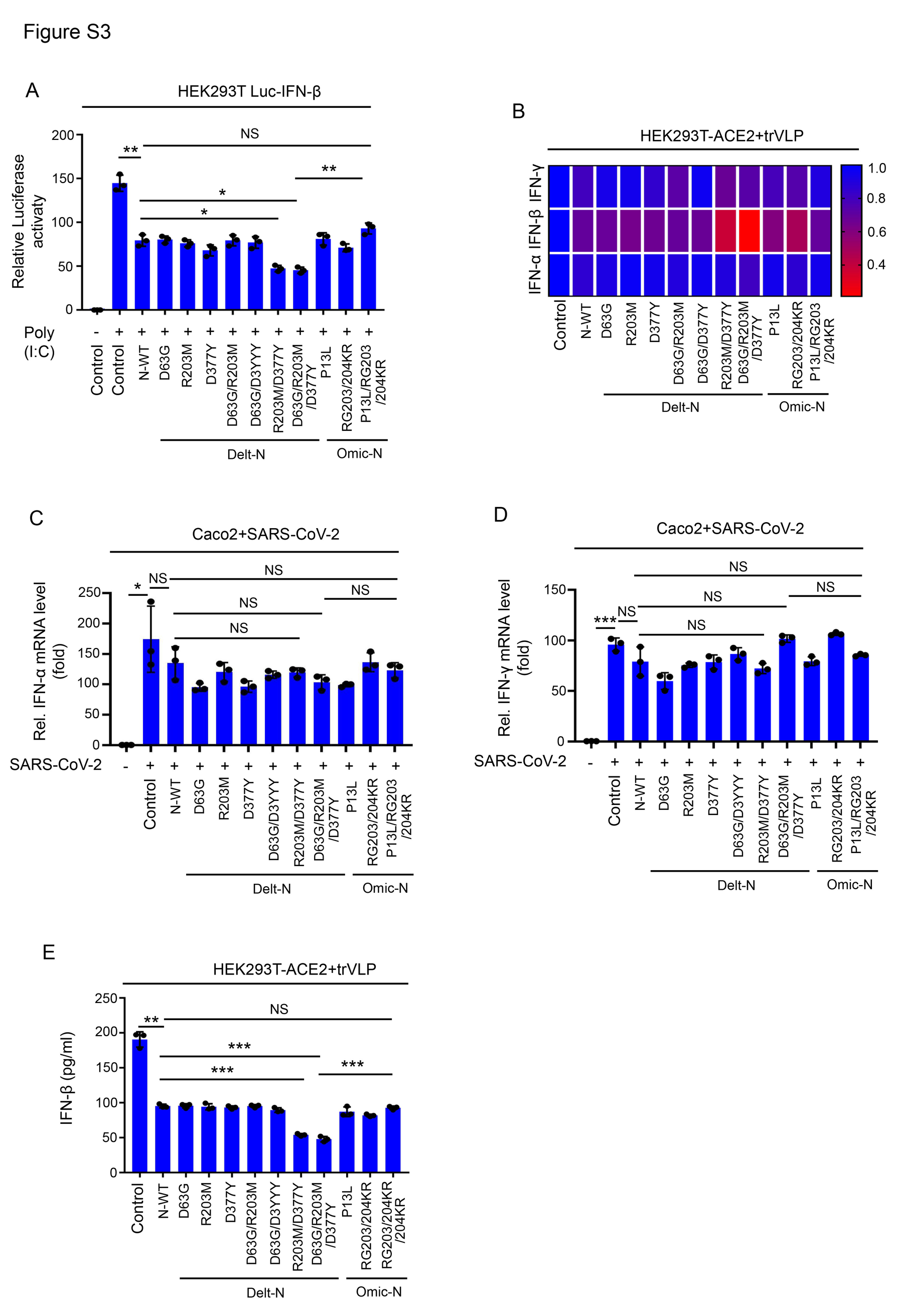

Supplement: S3 Fig — (A) HEK293T cells were transfected with IFN-β luciferase reporter pIFN-β-Luc, pPRL-TK and different mutated N protein for 24 h and then transfected with poly(I:C) (2 µg/mL) for 16 h. Cell lysates were harvested, IFN-β-Luc reporter activity was determined by dual luciferase reporter assays. (B) HEK293T-ACE2 cells were transfected with plasmids encoding different mutated N protein for 24 h, and then infected with SARS-CoV-2 GFP/ΔN (MOI = 5) for 2 h, washed and incubated for an additional 48 h. The total RNA in cells was extracted and RT-qPCR assays were conducted to determine IFN-α, IFN-β and IFN-γ. (C and D) Caco-2 cells were transfected with plasmids encoding different mutated N protein for 24 h, and then infected with SARS-CoV-2 (MOI = 0.5) for 2 h, washed and incubated for an additional 48 h. The total RNA in cells was extracted and RT-qPCR assays were conducted to determine IFN-α (C) and IFN-γ (D). (E) HEK293T-ACE2 cells were transfected with plasmids encoding different mutated N protein for 24 h, and then infected with SARS-CoV-2 GFP/ΔN (MOI = 0.5) for 2 h, washed and incubated for an additional 48h. Supernatants were analyzed by ELISA for IFN-β. Control means pcDNA3.1(+)-3 × flag empty plasmid (A–E). Data are representative of three independent experiments and one representative is shown. Error bars indicate SD of technical triplicates. Values are mean ± SEM. *P ≤ 0.05, **P ≤ 0.01, ***P ≤ 0.001, NS means No significant difference, two-tailed Student’s t-test. (TIF) [file ppat.1012886.s003.tif]

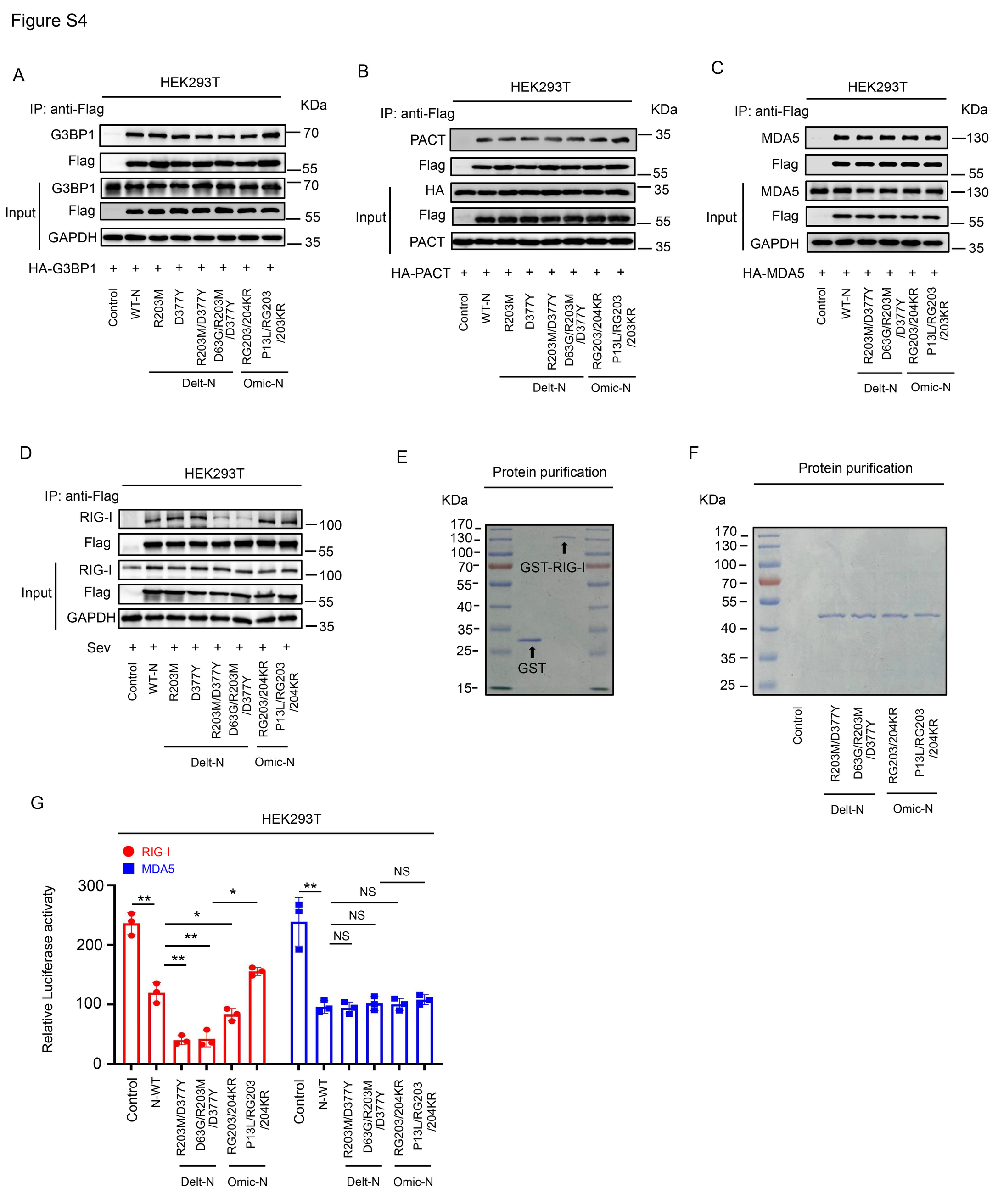

Supplement: S4 Fig — (A–C) HEK293T cells were co-transfected with plasmids encoding different mutated N protein and G3BP1 (A), PACT (B) or MDA5 (C) for 24 h. Cell lysates were immunoprecipitated using anti-Flag antibody, and analyzed using anti-Flag, anti-HA and anti-GAPDH antibody. Cell lysates (40 μg) was used as Input. (D) HEK293T cells were transfected with plasmids encoding different mutated N protein for 24 h and then infected with SeV (MOI = 0.1) for 16 h. Cell lysates were immunoprecipitated using anti-Flag antibody, and analyzed using anti-Flag, anti-RIG-I and anti-GAPDH antibody. Cell lysates (40 μg) was used as Input. (E) Coomassie blue staining analysis of the purified RIG-I-GST protein. (F) Coomassie blue staining analysis of the purified mutant N-His protein. (G) IFN-β luciferase reporter pIFN-β-Luc, pPRL-TK, different mutated N protein and RIG-I protein or pIFN-β-Luc, pPRL-TK, different mutated N protein and MDA5 protein for 24 h, and then infected with SeV (MOI = 0.1) for 16 h. Cell lysates were harvested, IFN-β-Luc reporter activity was determined by dual luciferase reporter assays. Control means pcDNA3.1(+)-3 × flag empty plasmid (A–D and G) or purified His-tagged empty protein (F). Data are representative of three independent experiments and one representative is shown. (TIF) [file ppat.1012886.s004.tif]

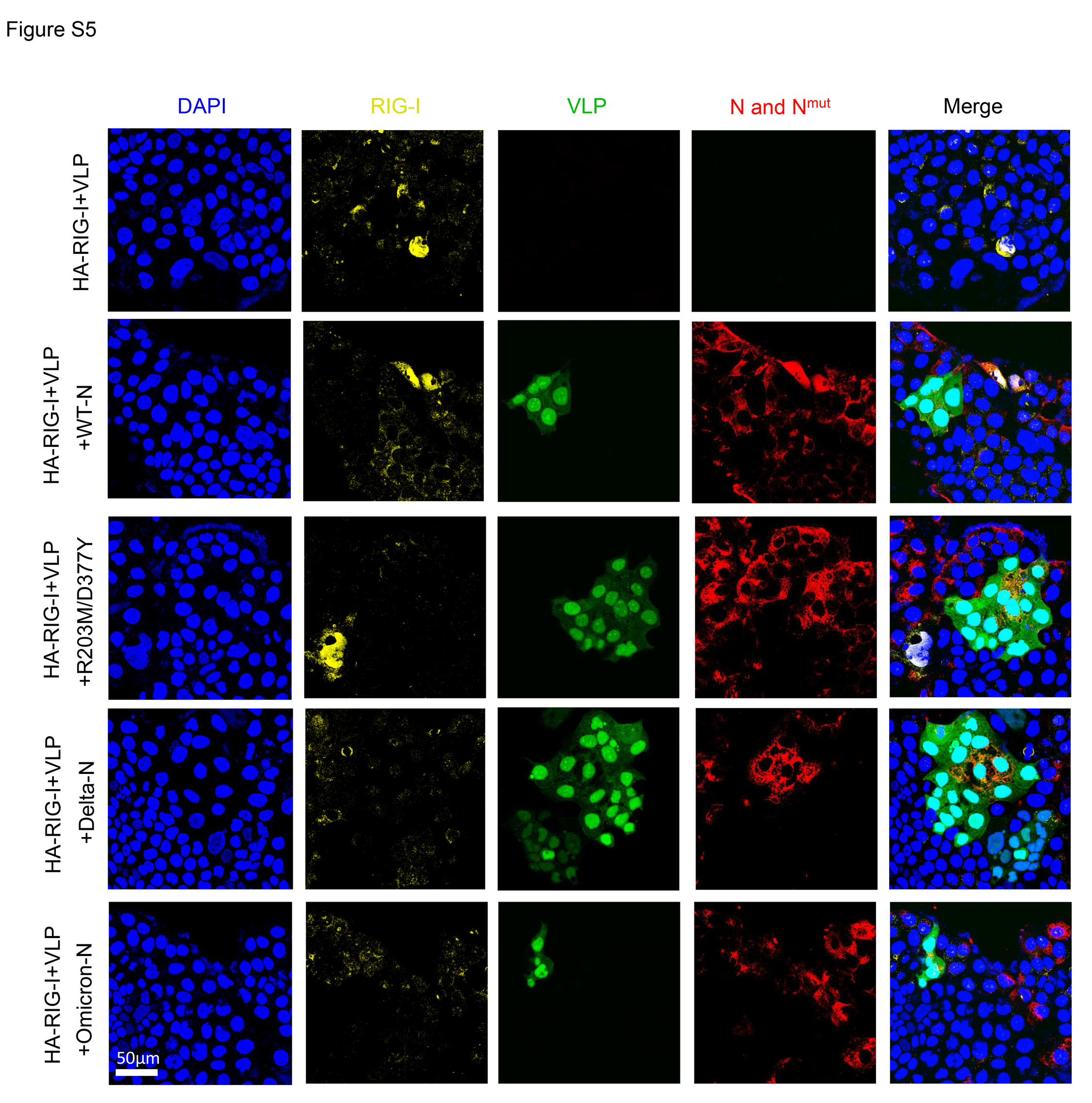

Supplement: S5 Fig — Caco-2 cells were co-transfected with plasmids encoding different mutated N protein and HA-tagged RIG-I protein for 24 h and then infected with SARS-CoV-2 GFP/ΔN (MOI = 5) for 48 h. Nucleus marker DAPI (blue), HA-tagged RIG-I protein (yellow), GFP- SARS-CoV-2 GFP/ΔN (green) and Flag- mutated N protein (red) were then visualized with confocal microscopy. Data are representative of three independent experiments and one representative is shown. (TIF) [file ppat.1012886.s005.tif]

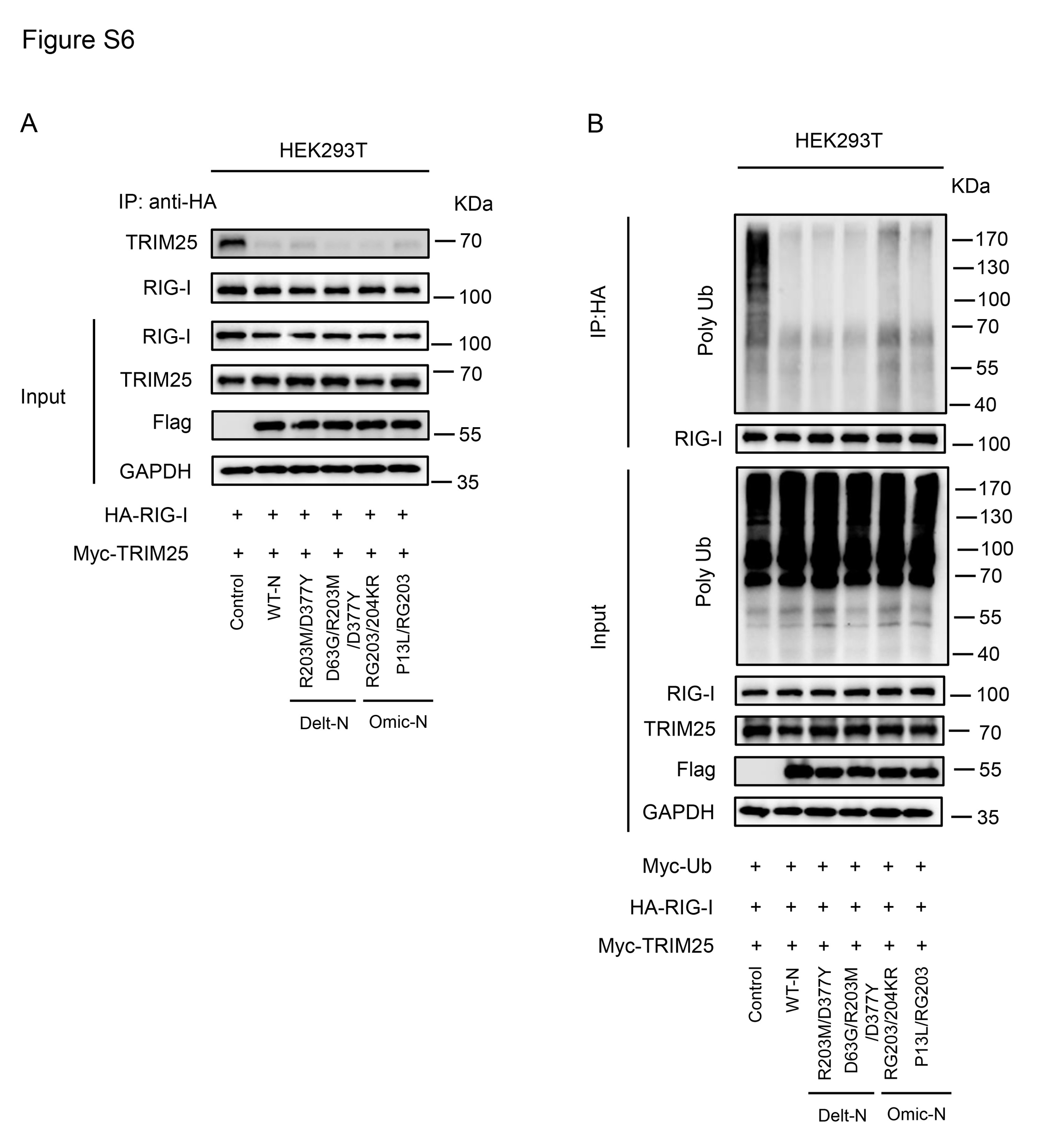

Supplement: S6 Fig — (A) HEK293T cells were co-transfected with plasmids encoding different mutated N protein with RIG-I and TRIM25 for 24 h. Cell lysates were immunoprecipitated using anti-HA antibody, and analyzed using anti-Flag, anti-HA, anti-Myc and anti-GAPDH antibody. Cell lysates (40 μg) was used as Input. (B) HEK293T cells were co-transfected with HA-RIG-I, Myc-TRIM25, different mutated N protein and Myc-Ubiquitin for 24 h. Cell lysates were immunoprecipitated using anti-HA antibody, and analyzed using anti-Ub, anti-HA, anti-GAPDH and anti-TRIM25 antibody. Cell lysates (40 μg) was used as Input. Control means pcDNA3.1(+)-3 × flag empty plasmid. Data are representative of three independent experiments and one representative is shown. (TIF) [file ppat.1012886.s006.tif]

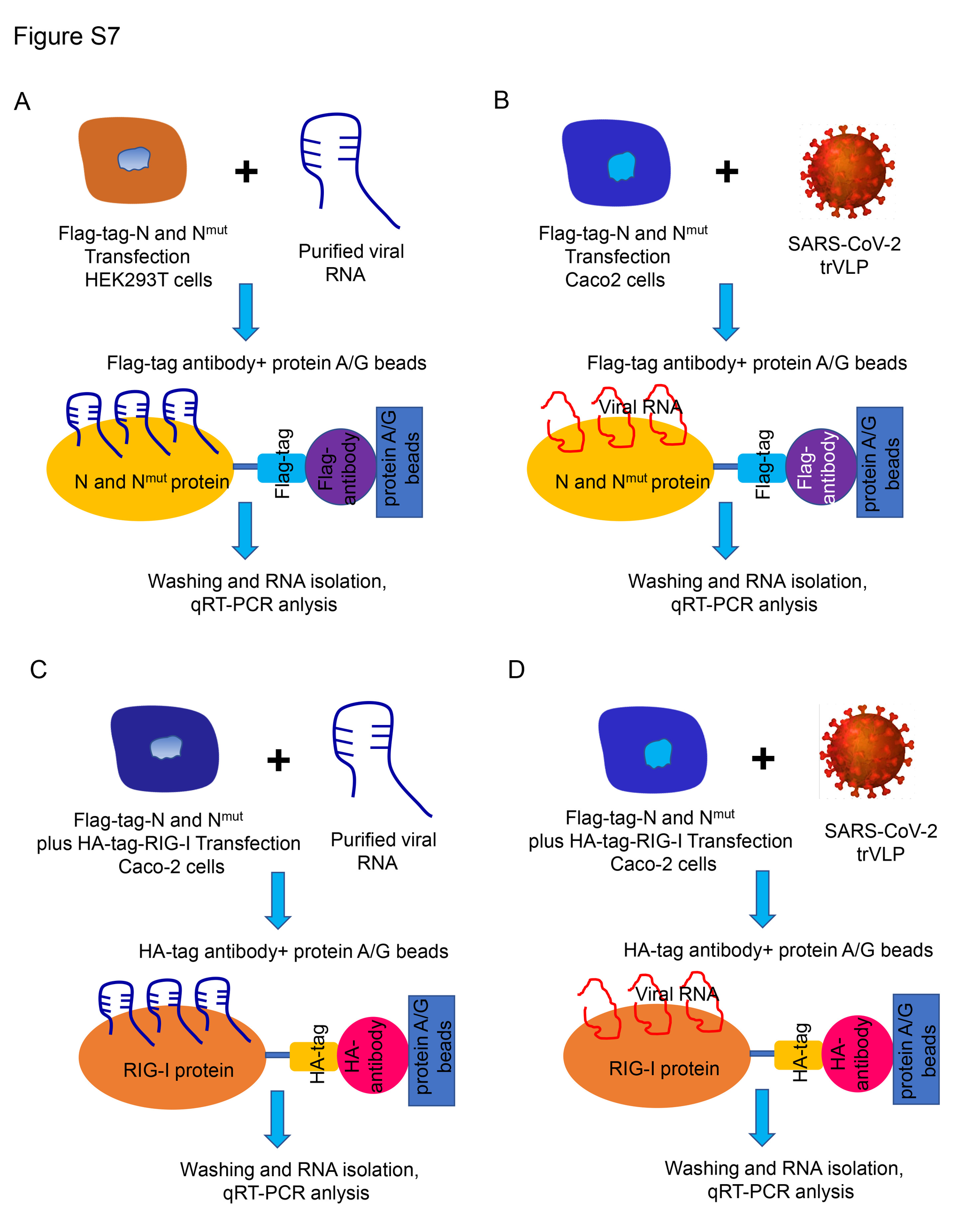

Supplement: S7 Fig — (A) HEK293T cells were transfected with plasmids encoding different mutated N protein for 24 h, Cell lysates were collected and incubated with purified RNA of SARS-CoV-2 GFP/ΔN VLP (10 µg) for 60 min, then the mixture was pulldown using Protein A/G MagBeads, washing and isolated RNAs were analyzed by RT-qPCR assay using specific viral N gene. (B) Caco-2 cells were transfected with plasmids encoding different mutated N protein for 24 h and then infected with SARS-CoV-2 GFP/ΔN (MOI = 0.5) for 2 h, washed and incubated for an additional 48 h. Cell lysates were pulldown using Protein A/G MagBeads, washing and isolated RNAs were analyzed by RT-qPCR assay using specific viral N gene. (C) Caco-2 cells were transfected with plasmids encoding different mutated N protein plus RIG-I protein for 24 h, Cell lysates were collected and incubated with purified RNA of SARS-CoV-2 GFP/ΔN VLP (10 µg) for 60 min, then the mixture was pulldown using Protein A/G MagBeads, washing and isolated RNAs were analyzed by RT-qPCR assay using specific viral N gene. (D) Caco-2 cells were transfected with plasmids encoding different mutated N protein plus RIG-I protein for 24 h and then infected with SARS-CoV-2 GFP/ΔN (MOI = 0.5) for 2 h, washed and incubated for an additional 48 h. Cell lysates were pulldown using Protein A/G MagBeads, washing and isolated RNAs were analyzed by RT-qPCR assay using specific viral N gene. (TIF) [file ppat.1012886.s007.tif]
