# Supplementary material for: The R203M and D377Y mutations of the nucleocapsid protein promote SARS-CoV-2 infectivity by impairing RIG-I-mediated antiviral signaling
Source: PLoS Pathog. 2025 Jan 22;21(1):e1012886. doi: 10.1371/journal.ppat.1012886 (PMC11771877; doi:10.1371/journal.ppat.1012886)
Supplement: S1 Table — (DOCX) [file ppat.1012886.s008.docx]

**S1 Table. qRT-PCR Primers used in this study.**

| **Name** | **Forward** 5’-3’ | **Reverse** 5’-3’ |
| --- | --- | --- |
| Human  *IFN-α* | TGAGACCCACAGCCTGGATA | CTGGAGCCTTCTGGAACTGG |
| Human  *IFN-β* | TGGGAGGCTTGAATACTGCCTCAA | TCCTTGGCCTTCAGGTAATGCAGA |
| Human  *IFN-γ* | CAGGTCATTCAGATGTAGCGGA | TCCTTGATGGTCTCCACACT |
| Human  *GAPDH* | AAGGCTGTGGGCAAGG | TGGAGGAGTGGGTGTCG |
| SARS-CoV-2  *ORF3a* | GCTTTGCTGGAAATGCCGTT | GGACTTGTTGTGCCATCACC |
| SARS-CoV-2  *NP* | ACCCGCAATCCTGCTAACAA | ACGAGAAGAGGCTTGACTGC |
| Mice  *IFN-α* | CTACTGGCCAACCTGCTCTC | CCTTCTTGATCTGCTGGGCA |
| Mice  *IFN-β* | CCGAGCAGAGATCTTCAGGAA | CCTGCAACCACCACTCATTCT |
| Mice  *IFN-γ* | GGTCAACAACCCACAGGTCC | CAGCGACTCCTTTTCCGCTT |
| VSV | TGATAGTACCGGAGGATTGACGAC | ATGGCGTATTTGAAAGTAGAA |
| Mice  *β-actin* | AGAGGGAAATCGTGCGTGAC | CAATAGTGATGACCTGGCCGT |
