# Supplementary material for: The R203M and D377Y mutations of the nucleocapsid protein promote SARS-CoV-2 infectivity by impairing RIG-I-mediated antiviral signaling
Source: PLoS Pathog. 2025 Jan 22;21(1):e1012886. doi: 10.1371/journal.ppat.1012886 (PMC11771877; doi:10.1371/journal.ppat.1012886)
Supplement: S2 Table — (DOCX) [file ppat.1012886.s009.docx]

**S2 Table. PCR Primers used in this study.**

| **Name** | **Forward** 5’-3’ | **Reverse** 5’-3’ |
| --- | --- | --- |
| *D63G* | CAACATGGCAAGGAAGGCCTTAAATTCCCTCG | CGAGGGAATTTAAGGCCTTCCTTGCCATGTTG |
| *R203M* | CTCCAGGCAGCAGTATGGGAACTTCTCCTGC | GCAGGAGAAGTTCCCATACTGCTGCCTGGAG |
| *D377Y* | CAAAAAGAAGAAGGCTTATGAAACTCAAGCC | GGCTTGAGTTTCATAAGCCTTCTTCTTTTTG |
| *P13L* | AATCAGCGAAATGCACTCCGCATTACGTTTG | CAAACGTAATGCGGAGTGCATTTCGCTGATT |
| *RG203/204KR* | ACTCCAGGCAGCAGTAAACGAACTTCTCCTGCT | AGCAGGAGAAGTTCGTTTACTGCTGCCTGGAGT |
