# Supplementary material for: The R203M and D377Y mutations of the nucleocapsid protein promote SARS-CoV-2 infectivity by impairing RIG-I-mediated antiviral signaling
Source: PLoS Pathog. 2025 Jan 22;21(1):e1012886. doi: 10.1371/journal.ppat.1012886 (PMC11771877; doi:10.1371/journal.ppat.1012886)
Supplement: S1 File — (DOCX) [file ppat.1012886.s010.docx]

**Attachment-**unprocessed images

**
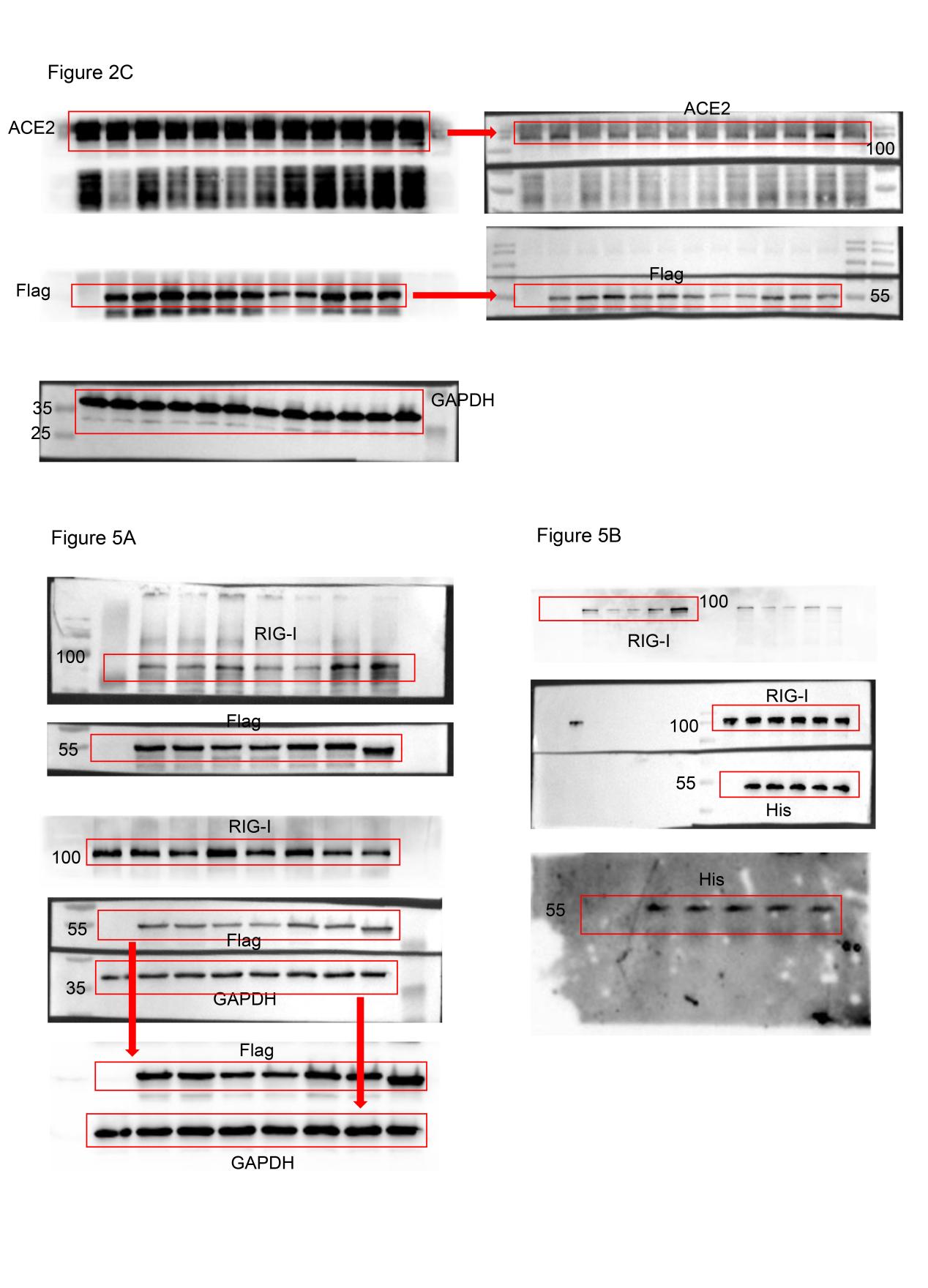
**

**
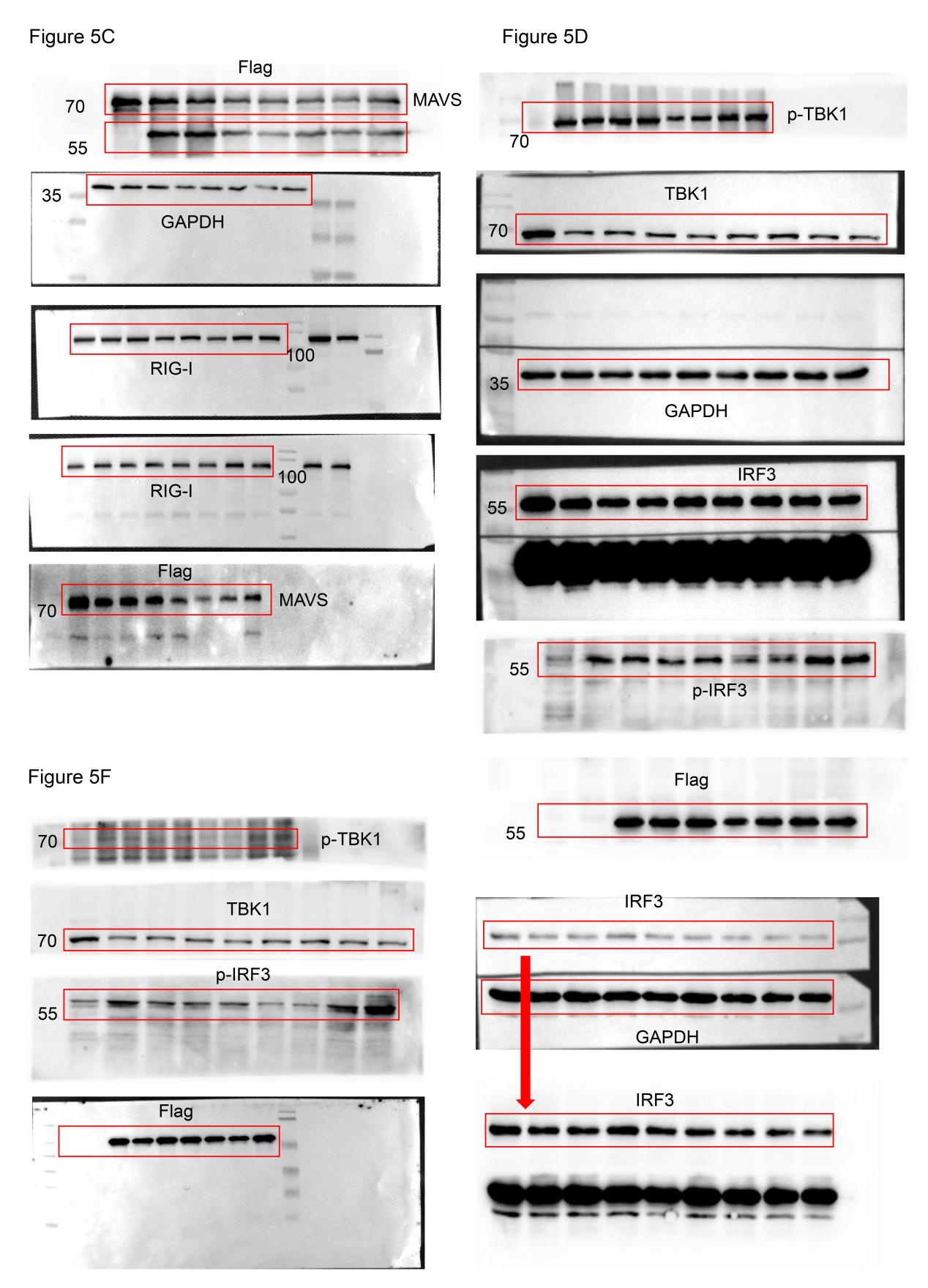
**

**
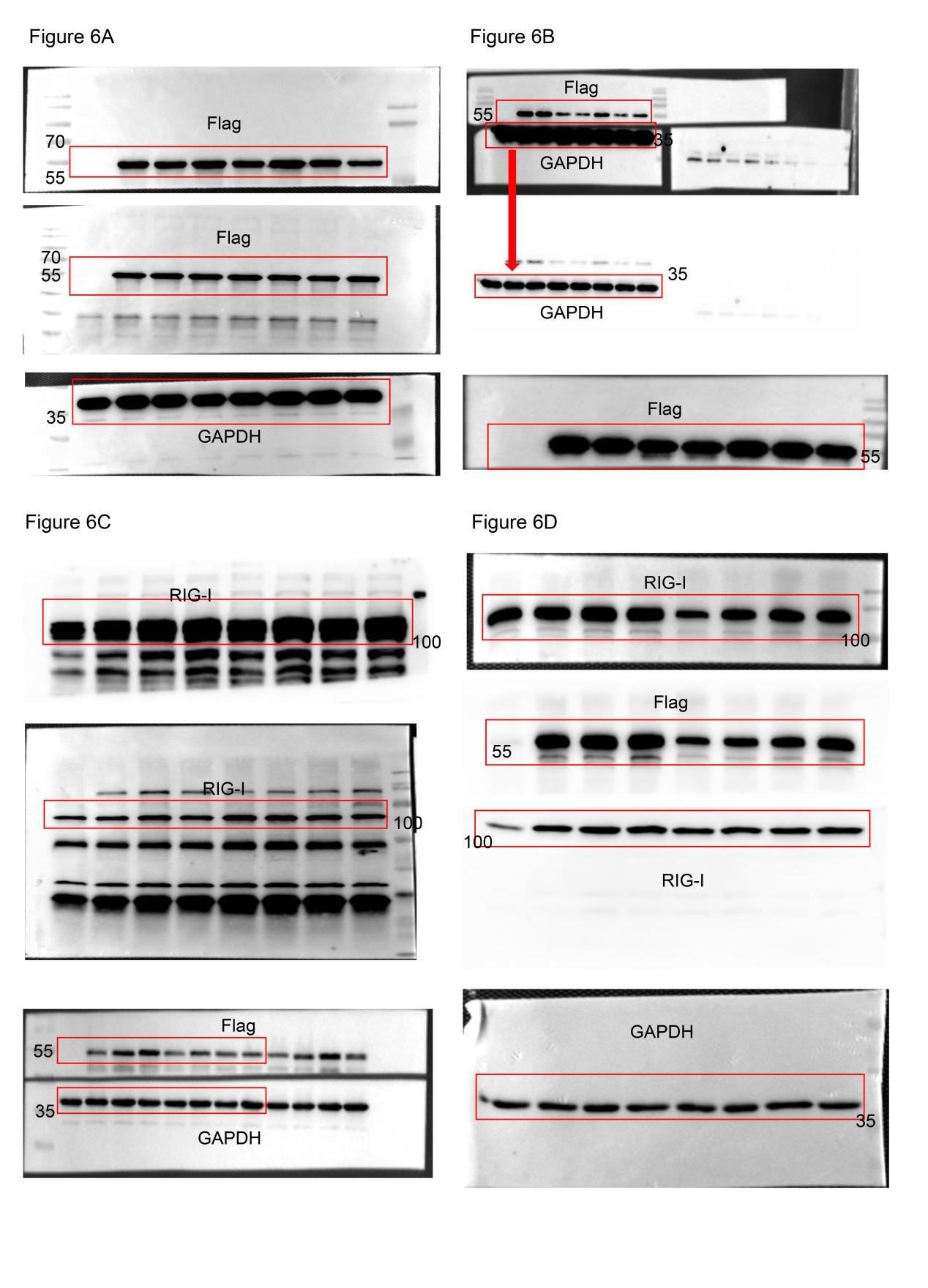
**

**
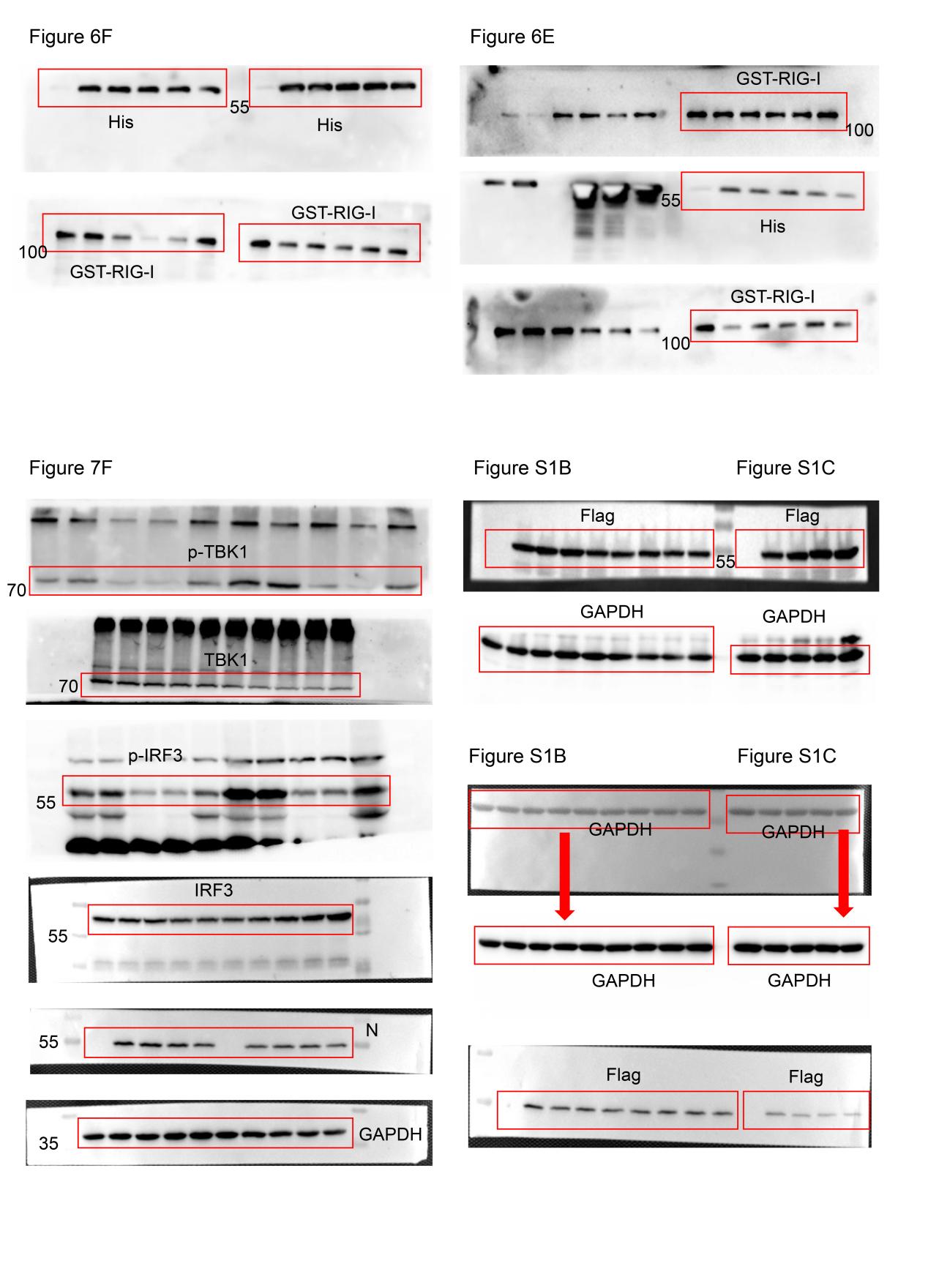
**

**
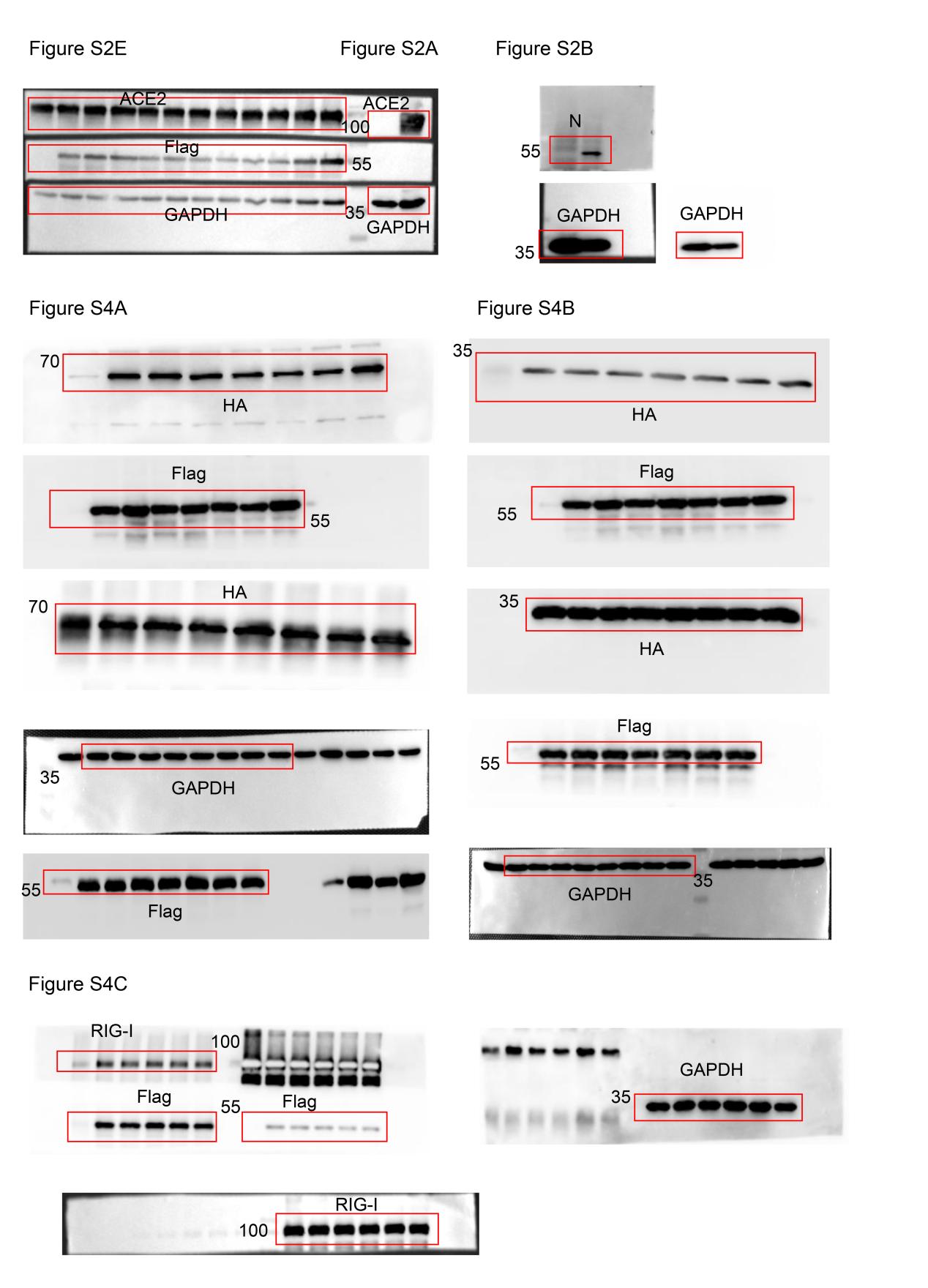
**

**
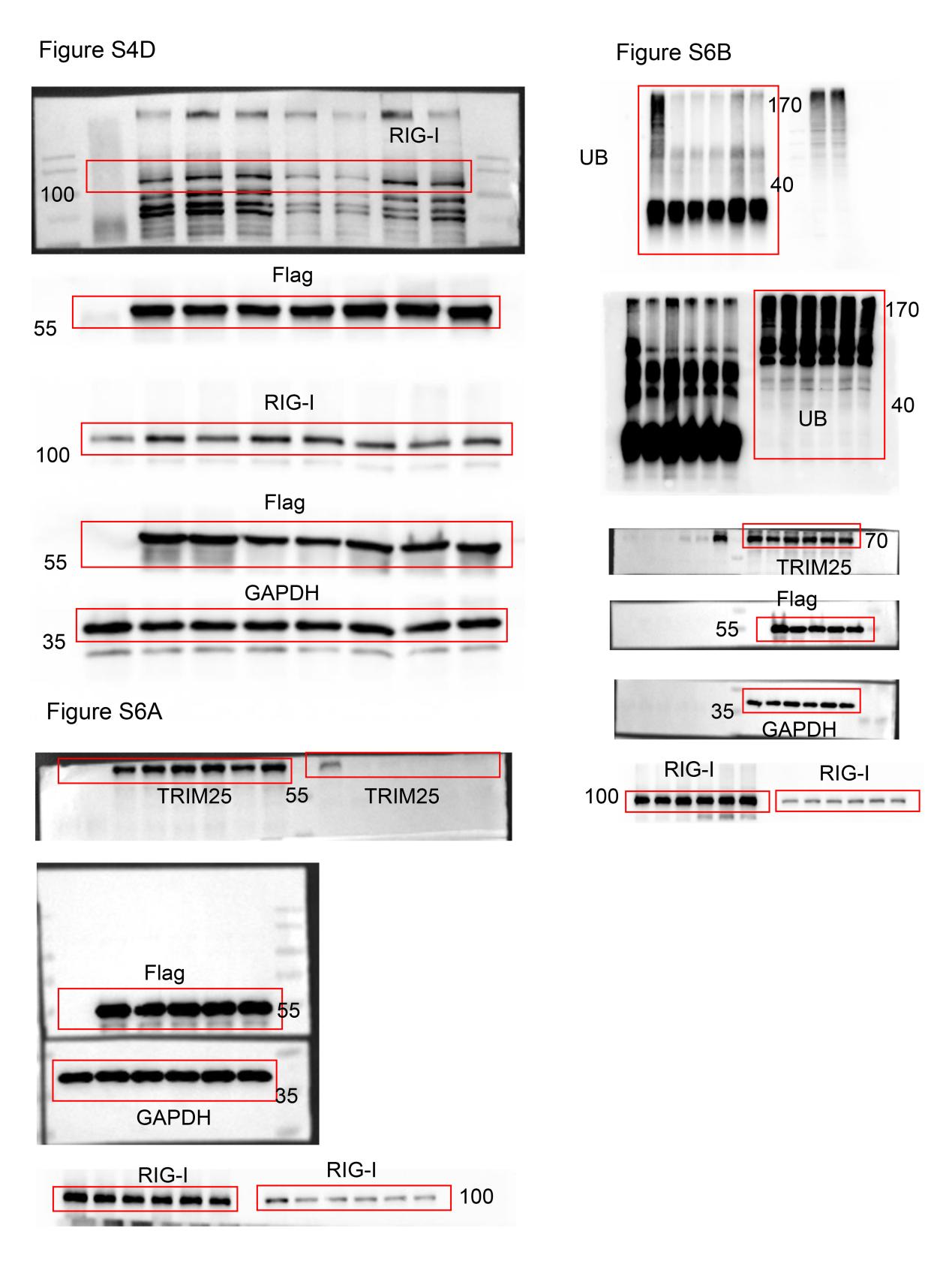
**

**Attachment-all raw data**

**Figure 2C**

| Control | 2.131214332 | 2.461163748 | 2.139677628 |
| --- | --- | --- | --- |
| WT-N | 6.129113135 | 6.182900893 | 6.182900893 |
| D63G | 6.205952904 | 6.205952904 | 6.200190076 |
| R203M | 6.002326786 | 6.127191973 | 6.050351911 |
| D377Y | 6.246293941 | 6.017695134 | 6.213637006 |
| D63G/R203M | 6.263583051 | 6.188663891 | 6.021537009 |
| D63G/D377Y | 6.257820117 | 5.988880011 | 6.07724614 |
| R203M/D377Y | 6.668914018 | 6.732306973 | 6.492182015 |
| D63G/R203M/D377Y | 6.607442048 | 6.676597963 | 6.803383976 |
| P13L | 6.303924025 | 6.188663891 | 6.134876135 |
| RG203/204KR | 6.323134072 | 6.280871898 | 6.325055059 |
| P13L/RG203/204KR | 5.98119602 | 5.904356008 | 5.919723978 |

**Figure 2E**

| Control | 2.0823796 | 2.359867721 | 2.171518762 |
| --- | --- | --- | --- |
| WT-N | 5.915881988 | 5.913961011 | 5.971591015 |
| R203M/D377Y | 6.738069969 | 6.64586204 | 6.620889012 |
| D63G/R203M/D377Y | 6.699650011 | 6.668914018 | 6.820673014 |
| RG203/204KR | 6.123350117 | 6.092613987 | 6.052272907 |
| P13L/RG203/204KR | 5.769885995 | 5.66038901 | 5.637336999 |

**Figure 3B**

| Control | 6.87E-08 | 0.000000177 | 1.77E-08 |
| --- | --- | --- | --- |
| WT-N | 0.0048 | 0.0122 | 0.0082 |
| D63G | 0.6355 | 0.8490 | 0.7990 |
| R203M | 0.6735 | 0.7580 | 0.7080 |
| D377Y | 0.7220 | 0.8355 | 0.7855 |
| D63G/R203M | 0.7125 | 0.8325 | 0.7825 |
| D63G/D377Y | 0.6115 | 0.7705 | 0.8205 |
| R203M/D377Y | 2.3640 | 2.5090 | 2.6090 |
| D63G/R203M/D377Y | 2.6140 | 2.5200 | 2.7200 |
| P13L | 0.6070 | 0.7600 | 0.8100 |
| RG203/204KR | 1.1960 | 1.1267 | 1.2600 |
| P13L/RG203/204KR | 0.5060 | 0.3840 | 0.4340 |

**Figure 3C**

| Control | 2.158362492 | 2.296935334 | 2.241139637 |
| --- | --- | --- | --- |
| WT-N | 7.383526 | 7.387368 | 7.327817 |
| D63G | 8.584151 | 8.749357 | 8.73591 |
| R203M | 8.693648 | 8.564941 | 8.77433 |
| D377Y | 8.970272 | 8.770488 | 8.83196 |
| D63G/R203M | 8.916484 | 8.770488 | 8.628334 |
| D63G/D377Y | 8.795461 | 8.574546 | 8.818513 |
| R203M/D377Y | 9.250738 | 9.135478 | 9.325657 |
| D63G/R203M/D377Y | 9.425549 | 9.264185 | 9.306447 |
| P13L | 8.814671 | 8.849249 | 8.810829 |
| RG203/204KR | 8.929931 | 8.920326 | 9.035586 |
| P13L/RG203/204KR | 8.265265 | 8.534205 | 8.345947 |

**Figure 3D**

| Control | 0.000000494 | 0.00000044 | 0.00000074 |
| --- | --- | --- | --- |
| WT-N | 0.0121 | 0.0123 | 0.0103 |
| D63G | 1.0790 | 0.8876 | 1.1076 |
| R203M | 0.8672 | 0.9864 | 1.2964 |
| D377Y | 1.0170 | 0.8562 | 1.4562 |
| D63G/R203M | 1.2430 | 0.9486 | 0.8286 |
| D63G/D377Y | 1.1980 | 0.9483 | 1.5083 |
| R203M/D377Y | 4.1820 | 4.6198 | 3.8198 |
| D63G/R203M/D377Y | 4.8960 | 5.7716 | 4.3716 |
| P13L | 1.0320 | 1.1200 | 0.9200 |
| RG203/204KR | 1.5634 | 1.6138 | 2.0138 |
| P13L/RG203/204KR | 0.7710 | 0.5802 | 0.4802 |

**Figure 3E**

| Control | 2.100659289 | 2.185873203 | 2.403835574 |
| --- | --- | --- | --- |
| WT-N | 7.022378 | 6.807226 | 6.766885 |
| D63G | 8.612966 | 8.643702 | 8.647544 |
| R203M | 8.732068 | 8.722463 | 8.664833 |
| D377Y | 8.603361 | 8.643702 | 8.611045 |
| D63G/R203M | 8.457365 | 8.536126 | 8.468891 |
| D63G/D377Y | 8.557257 | 8.599519 | 8.81275 |
| R203M/D377Y | 8.935694 | 9.089374 | 9.148925 |
| D63G/R203M/D377Y | 9.127794 | 9.264185 | 9.210397 |
| P13L | 8.630255 | 8.572625 | 8.614887 |
| RG203/204KR | 8.737831 | 8.826197 | 8.845407 |
| P13L/RG203/204KR | 8.173057 | 8.215319 | 8.04435 |

**Figure 4A**

|  | **Original** | | | **Delta** | | | **Omicron** | | |
| --- | --- | --- | --- | --- | --- | --- | --- | --- | --- |
| 0 | 215.1249 | 286.3190 | 269.2352 | 299.9244 | 361.6993 | 352.9557 | 219.3985 | 273.1878 | 256.5737 |
| 6 | 3252160.27 | 3318294.687 | 3322588.044 | 2074644.539 | 2264676.418 | 2335667.217 | 680266.6738 | 757339.4521 | 2218891.395 |
| 12 | 168284211.1 | 169882159.8 | 168993882.9 | 173412620.8 | 188334593.6 | 189227959 | 112608764.8 | 118886211.8 | 114192434.4 |
| 24 | 250279700.7 | 236355856.2 | 240518790.9 | 268055144.8 | 288630992.4 | 279017554.7 | 210522253.5 | 213011158 | 178468820.5 |
| 48 | 239579787.7 | 231763122.8 | 160596845.3 | 317505814.4 | 334247502.8 | 343115568 | 163293034.1 | 169435675.2 | 154689393.9 |
| 72 | 139498151.1 | 135752051.7 | 139478380.6 | 207870894 | 220906522.2 | 213203802.1 | 108749835 | 115146489.5 | 122038710.5 |

**Figure 4B**

|  | **Original** | | | **Delta** | | | **Omicron** | | |
| --- | --- | --- | --- | --- | --- | --- | --- | --- | --- |
| 0 | 0.846309508 | 0.995488397 | 0.963706098 | 1.519647258 | 1.237302422 | 1.220402817 | 1.055555402 | 1.78792326 | 0.529870929 |
| 6 | 1.157283349 | 0.879659709 | 2.628172885 | 1.222164037 | 2.172764068 | 1.769265127 | 1.640094135 | 2.217608203 | 0.754587659 |
| 12 | 28.72617275 | 33.96326667 | 37.11517105 | 16.54041794 | 21.39597505 | 18.47684078 | 75.5380688 | 75.23778747 | 67.17094175 |
| 24 | 145.4612772 | 113.4580587 | 119.0839386 | 32.51577677 | 37.55849379 | 28.69492941 | 172.2241133 | 154.0465113 | 111.6574594 |
| 48 | 158.1036424 | 144.8997039 | 159.3586156 | 41.75853085 | 31.47958437 | 29.53522124 | 215.0002809 | 270.8601417 | 204.3648604 |
| 72 | 901.9064489 | 1154.836112 | 727.21789 | 125.1339364 | 121.1493452 | 142.8687575 | 1177.676472 | 879.6366769 | 1508.917252 |

**Figure 4C**

| Control | 0 | 0 | 0 |
| --- | --- | --- | --- |
| Control | 485 | 430 | 465 |
| WT-N | 131 | 158 | 168 |
| D63G | 157 | 149 | 169 |
| R203M | 173 | 166 | 186 |
| D377Y | 151 | 177 | 166 |
| D63G/R203M | 137 | 122 | 158 |
| D63G/D377Y | 148 | 121 | 159 |
| R203M/D377Y | 72 | 85 | 61 |
| D63G/R203M/D377Y | 78 | 57 | 65 |
| P13L | 167 | 180 | 146 |
| RG203/204KR | 101 | 147 | 135 |
| P13L/RG203/204KR | 198 | 162 | 178 |

**Figure 4D**

|  | **IFN-α** | **IFN-β** | **IFN-****γ** |
| --- | --- | --- | --- |
| Control | 1 | 1 | 1 |
| WT-N | 0.7887 | 0.6231 | 0.7096 |
| D63G | 0.8263 | 0.6322 | 0.7354 |
| R203M | 0.6197 | 0.5967 | 0.7292 |
| D377Y | 0.7465 | 0.5603 | 0.7169 |
| D63G/R203M | 0.7512 | 0.6653 | 0.8231 |
| D63G/D377Y | 0.7793 | 0.6719 | 0.7031 |
| R203M/D377Y | 0.7371 | 0.2099 | 0.6462 |
| D63G/R203M/D377Y | 0.7042 | 0.1496 | 0.6362 |
| P13L | 0.8404 | 0.7496 | 0.8154 |
| RG203/204KR | 0.7465 | 0.5868 | 0.6469 |
| P13L/RG203/204KR | 0.9765 | 0.743 | 0.8923 |

**Figure 4E**

| Mock | 1 | 0.8468849 | 0.926082365 |
| --- | --- | --- | --- |
| Control | 713.8331573 | 1044.350581 | 753.9598733 |
| WT-N | 110.4540655 | 87.22280887 | 107.9197466 |
| D63G | 95.24815206 | 115.9450898 | 102.4287223 |
| R203M | 105.1742344 | 113.4107709 | 106.4413939 |
| D377Y | 104.3294615 | 107.2861668 | 89.12354805 |
| D63G/D377Y | 107.4973601 | 136.8532207 | 106.2302006 |
| R203M/D377Y | 19.00739176 | 20.6969377 | 15.73389652 |
| D63G/R203M/D377Y | 16.47307286 | 29.25026399 | 32.8405491 |
| P13L | 95.03695882 | 107.0749736 | 97.57127772 |
| RG203/204KR | 61.93241816 | 62.24920803 | 63.51636748 |
| P13L/RG203/204KR | 266.1034847 | 257.655755 | 215.4171067 |

**Figure 4F**

| Control | 338.4808 | 353.1923 | 365.4808 |
| --- | --- | --- | --- |
| WT-N | 155.7115 | 162.9808 | 159.5769 |
| D63G | 168.8077 | 161.4808 | 168.3462 |
| R203M | 162.5769 | 160.7885 | 165.5192 |
| D377Y | 156.6923 | 157.2115 | 170.4231 |
| D63G/R203M | 152.7692 | 159.5769 | 152.6538 |
| D63G/D377Y | 170.0192 | 178.6731 | 177.1731 |
| R203M/D377Y | 69.7308 | 64.3462 | 68.0000 |
| D63G/R203M/D377Y | 63.1539 | 80.7692 | 65.0769 |
| P13L | 162.1731 | 178.9615 | 159.0000 |
| RG203/204KR | 132.6923 | 142.9615 | 138.0577 |
| P13L/RG203/204KR | 162.9808 | 162.1731 | 178.9615 |

**Figure 4G**

|  | **Untreated** | | | **Anifrolumab** | | |
| --- | --- | --- | --- | --- | --- | --- |
| Control | 2.19914398 | 2.223194977 | 2.222551855 | 2.103157362 | 2.246788981 | 2.00483618 |
| WT-N | 4.868839177 | 4.815755722 | 4.73547022 | 5.735113066 | 5.754489476 | 5.487174841 |
| R203M/D377Y | 5.543013065 | 5.754489476 | 5.448754841 | 5.735113066 | 5.831329476 | 5.640854841 |
| D63G/R203M/D377Y | 5.682766752 | 5.770747855 | 5.744426513 | 5.784225328 | 5.766092135 | 5.795533711 |
| RG203/204KR | 5.160624615 | 5.156895104 | 5.148655322 | 5.484931655 | 5.684420695 | 5.56274002 |
| P13L/RG203/204KR | 4.517405083 | 4.543647739 | 4.462547608 | 5.720934207 | 5.622067829 | 5.697262567 |

**Figure 4H**

|  | **Untreated** | | | **Anifrolumab** | | |
| --- | --- | --- | --- | --- | --- | --- |
| Control | 7.511385534 | 7.483680824 | 7.493445272 | 8.341437354 | 8.298535426 | 8.293059767 |
| WT-N | 8.07243917 | 8.116850426 | 8.025812055 | 8.311185405 | 8.480144676 | 8.284366299 |
| R203M/D377Y | 8.428224147 | 8.460342963 | 8.445631528 | 8.48634899 | 8.421155839 | 8.506543334 |
| D63G/R203M/D377Y | 8.488732612 | 8.438030735 | 8.509832819 | 8.46388983 | 8.457057603 | 8.459887696 |
| RG203/204KR | 8.248447973 | 8.230836359 | 8.216903231 | 8.42987758 | 8.419665992 | 8.403828151 |
| P13L/RG203/204KR | 7.743238836 | 7.730514393 | 7.75856834 | 8.380183724 | 8.486028715 | 8.343347377 |

**Figure 5E**

| Control | 5 | 8 | 6 |
| --- | --- | --- | --- |
| Control | 80 | 85 | 92 |
| WT-N | 55 | 68 | 61 |
| R203M/D377Y | 30 | 35 | 38 |
| D63G/R203M/D377Y | 32 | 28 | 26 |
| P13L/RG203/204KR | 70 | 75 | 79 |

**Figure 6A**

| Control | 2.43 | 2.12 | 1.86 |
| --- | --- | --- | --- |
| WT-N | 23.4 | 21.8 | 25.9 |
| R203M | 26.53 | 24.97 | 22.85 |
| D377Y | 27.84 | 22.48 | 25.49 |
| R203M/D377Y | 67.61 | 69.37 | 70.25 |
| D63G/R203M/D377Y | 76.13 | 73.57 | 72.48 |
| RG203/204KR | 48.37 | 44.21 | 46.92 |
| P13L/RG203/204KR | 19.55 | 17.23 | 16.49 |

**Figure 6B**

| Control | 1.58 | 1.14 | 0.86 |
| --- | --- | --- | --- |
| WT-N | 30.21 | 28.84 | 32.92 |
| R203M | 29.24 | 26.75 | 32.15 |
| D377Y | 30.84 | 32.42 | 35.29 |
| R203M/D377Y | 61.83 | 58.29 | 57.26 |
| D63G/R203M/D377Y | 63.54 | 60.24 | 59.21 |
| RG203/204KR | 43.57 | 45.32 | 40.39 |
| P13L/RG203/204KR | 23.55 | 27.29 | 22.96 |

**Figure 6C**

| Control | 69.6 | 72.9 | 65.3 |
| --- | --- | --- | --- |
| WT-N | 43.68 | 47.84 | 47.19 |
| R203M | 42.25 | 48.36 | 46.41 |
| D377Y | 45.76 | 40.82 | 43.03 |
| R203M/D377Y | 23.2 | 26.1 | 20.4 |
| D63G/R203M/D377Y | 19.5 | 21.4 | 17.5 |
| RG203/204KR | 29.5 | 26.3 | 32.2 |
| P13L/RG203/204KR | 55.245 | 63.3 | 55.8 |

**Figure 6D**

| Control | 72.8 | 78.8 | 83.6 |
| --- | --- | --- | --- |
| WT-N | 53.2 | 50.3 | 48.3 |
| R203M | 46.9 | 50.3 | 53.2 |
| D377Y | 48.2 | 51.3 | 54.2 |
| R203M/D377Y | 24.3 | 27.3 | 21.2 |
| D63G/R203M/D377Y | 16.8 | 19.3 | 23.1 |
| RG203/204KR | 35.2 | 31.9 | 38.2 |
| P13L/RG203/204KR | 59.4 | 62.9 | 66.3 |

**Figure 6E**

| Control | 0.680570312 | 0.683587323 | 0.760147725 |
| --- | --- | --- | --- |
| WT-N | 0.492765166 | 0.459096519 | 0.447072545 |
| R203M/D377Y | 0.178159916 | 0.221278389 | 0.247151926 |
| D63G/R203M/D377Y | 0.12125035 | 0.187042802 | 0.150595502 |
| RG203/204KR | 0.259474689 | 0.366381048 | 0.285994051 |
| P13L/RG203/204KR | 0.651122945 | 0.585543278 | 0.6340697 |

**Figure 7A**

**IFN-β**

| **Control** | **WT-N** | **R203M/D377Y** | **Delta-N** | **Omicron-N** | **Control+VSV** | **WT-N+VSV** | **R203M/D377Y+VSV** | **Delta-N+VSV** | **Omicron-N+VSV** |
| --- | --- | --- | --- | --- | --- | --- | --- | --- | --- |
| 3.063115994 | 1.128935061 | 0.688963758 | 0.298851357 | 1.76846786 | 264.9158946 | 156.7693809 | 56.07907297 | 55.11710068 | 169.7056275 |
| 3.000077979 | 0.773782497 | 0.629596751 | 0.348082213 | 1.921855677 | 225.8765351 | 144.2572483 | 46.83095637 | 50.36774004 | 149.7996659 |
| 4.773342972 | 2.732080514 | 0.497407443 | 0.187830077 | 1.895396922 | 232.2267888 | 167.3072542 | 42.35405205 | 39.79259542 | 190.5275996 |
| 5.045509635 | 2.514026749 | 0.500867185 | 0.229648617 | 2.045565877 | 172.3730709 | 168.4709682 | 36.87134387 | 40.06937481 | 193.1872658 |
| 1.895396922 | 0.836204722 | 0.493971599 | 0.282730612 | 1.397162668 | 193.522324 | 131.0912966 | 40.90919083 | 37.04786989 | 184.0375301 |
| 2.045565877 | 0.807720519 | 0.43906304 | 0.290679251 | 1.51834538 | 185.6390217 | 111.0009409 | 37.12780435 | 32.92972801 | 204.2024251 |

**IFN-α**

| **Control** | **WT-N** | **R203M/D377Y** | **Delta-N** | **Omicron-N** | **Control+VSV** | **WT-N+VSV** | **R203M/D377Y+VSV** | **Delta-N+VSV** | **Omicron-N+VSV** |
| --- | --- | --- | --- | --- | --- | --- | --- | --- | --- |
| 1.689538431 | 2.418590742 | 1.829823081 | 1.680821669 | 2.792711737 | 104.5895398 | 66.19244982 | 86.73814962 | 115.8367872 | 121.6080364 |
| 2.109102437 | 2.957066457 | 2.206414994 | 1.669211386 | 2.237157249 | 63.05744288 | 78.71653228 | 106.4149667 | 130.3231603 | 89.02229331 |
| 2.763753922 | 2.418590742 | 1.961155819 | 3.292461719 | 3.056046863 | 119.311952 | 91.05048277 | 104.5895398 | 72.80419537 | 98.09412043 |
| 2.439573868 | 1.807714277 | 1.496616064 | 3.504398081 | 2.812136583 | 81.4924648 | 85.54399989 | 103.1496247 | 83.63005946 | 89.64149233 |
| 4.981669002 | 3.019200709 | 2.792784328 | 5.274983813 | 3.131175051 | 171.0879998 | 147.9119456 | 140.4153904 | 153.9106959 | 168.4404426 |
| 6.090789172 | 2.778231207 | 3.014051705 | 2.787876527 | 2.688318674 | 126.9920842 | 148.9407546 | 122.2386972 | 154.9812294 | 136.6334269 |

**IFN-γ**

| **Control** | **WT-N** | **R203M/D377Y** | **Delta-N** | **Omicron-N** | **Control+VSV** | **WT-N+VSV** | **R203M/D377Y+VSV** | **Delta-N+VSV** | **Omicron-N+VSV** |
| --- | --- | --- | --- | --- | --- | --- | --- | --- | --- |
| 1.210693211 | 1.002313162 | 2.025577568 | 1.043068087 | 1.435352654 | 24.05614116 | 26.10773215 | 27.10076043 | 20.82524305 | 20.61801206 |
| 1.032279275 | 0.860551437 | 1.373953647 | 1.206504531 | 1.198645662 | 23.56107293 | 29.98990898 | 20.65377133 | 29.86246656 | 14.08251725 |
| 3.022690642 | 1.38831345 | 1.739090889 | 3.708493739 | 1.767126401 | 36.51105441 | 27.72846472 | 18.23114584 | 18.38250444 | 25.29527177 |
| 3.424357518 | 0.713672127 | 1.633915453 | 1.329453515 | 2.086963312 | 42.82144065 | 10.95331632 | 28.05646668 | 32.00579919 | 20.83295861 |
| 1.262106219 | 1.002313162 | 1.482809572 | 2.568334895 | 3.141394737 | 59.23316758 | 32.86165724 | 25.07770349 | 23.10705394 | 37.94488016 |
| 1.297588855 | 1.167427804 | 1.611420856 | 2.515479327 | 3.055493133 | 50.50426543 | 34.77472485 | 20.94208723 | 21.11595261 | 33.49405941 |

**Figure 7B**

| **Control** | **WT-N** | **R203M/D377Y** | **Delta-N** | **Omicron-N** | **Control+VSV** | **WT-N+VSV** | **R203M/D377Y+VSV** | **Delta-N+VSV** | **Omicron-N+VSV** |
| --- | --- | --- | --- | --- | --- | --- | --- | --- | --- |
| 17.301458 | 8.830853 | 4.056512 | 13.451183 | 18.841568 | 465.64148 | 199.174448 | 82.742132 | 51.4919 | 229.402607 |
| 21.305744 | 15.145304 | 13.297172 | 6.982721 | 21.921788 | 440.43968 | 188.869712 | 88.45454 | 56.728274 | 263.621051 |
| 31.162448 | 2.824424 | 0.514259 | 3.594479 | 6.520688 | 451.36046 | 187.077584 | 80.894 | 62.580692 | 253.092299 |
| 27.466184 | 7.598765 | 3.594479 | 14.067227 | 7.444754 | 450.5204 | 262.794992 | 51.77192 | 91.226738 | 261.64691 |
| 14.991293 | 11.603051 | 3.286457 | 3.132446 | 14.683271 | 410.19752 | 199.174448 | 105.53576 | 60.424538 | 278.756132 |
| 4.980578 | 28.544261 | 3.440468 | 4.980578 | 28.39025 | 460.60112 | 188.869712 | 83.13416 | 62.27267 | 240.589406 |

**Figure 7C**

**IFN-β**

| **Control** | **WT-N** | **R203M/D377Y** | **Delta-N** | **Omicron-N** | **Control+VSV** | **WT-N+VSV** | **R203M/D377Y+VSV** | **Delta-N+VSV** | **Omicron-N+VSV** |
| --- | --- | --- | --- | --- | --- | --- | --- | --- | --- |
| 1.105730653 | 1.021038665 | 0.635075491 | 0.463829558 | 3.71921977 | 24.847004 | 15.55965936 | 8.556931217 | 6.276509639 | 19.00435735 |
| 1.031683179 | 0.933057244 | 0.609205132 | 0.507565764 | 3.877159268 | 21.18542257 | 15.45218095 | 9.56054919 | 6.543046396 | 23.23549275 |
| 1.113421618 | 1.035291833 | 0.771105413 | 0.493686352 | 3.82378127 | 26.26373148 | 16.10836957 | 9.29911547 | 6.726996468 | 18.74271875 |
| 0.787307977 | 0.732061876 | 0.545253866 | 0.349088967 | 2.703821666 | 18.57126263 | 11.39033735 | 6.575467608 | 4.75670482 | 13.25310352 |

**IFN-α**

| **Control** | **WT-N** | **R203M/D377Y** | **Delta-N** | **Omicron-N** | **Control+VSV** | **WT-N+VSV** | **R203M/D377Y+VSV** | **Delta-N+VSV** | **Omicron-N+VSV** |
| --- | --- | --- | --- | --- | --- | --- | --- | --- | --- |
| 1.071773463 | 1.314976939 | 1.164733586 | 0.911722489 | 2.158456473 | 19.31872658 | 18.72107881 | 19.55312676 | 22.16059944 | 20.68751217 |
| 1.086734863 | 1.270183997 | 1.164733586 | 0.937354497 | 2.114036081 | 21.58456473 | 18.08337014 | 20.95652237 | 19.42610826 | 22.47460615 |
| 1.132883885 | 1.244043989 | 1.132883885 | 0.92444966 | 2.219138944 | 20.84931522 | 19.51608367 | 20.66800773 | 21.11102909 | 19.20983273 |
| 0.757858283 | 0.929829111 | 0.823591017 | 0.644685154 | 1.526259209 | 13.66040257 | 13.23780178 | 13.82614852 | 15.66991014 | 14.62828014 |

**IFN-γ**

| **Control** | **WT-N** | **R203M/D377Y** | **Delta-N** | **Omicron-N** | **Control+VSV** | **WT-N+VSV** | **R203M/D377Y+VSV** | **Delta-N+VSV** | **Omicron-N+VSV** |
| --- | --- | --- | --- | --- | --- | --- | --- | --- | --- |
| 0.987943197 | 0.964289002 | 0.994814886 | 1.164061004 | 1.178978573 | 11.99555576 | 15.23340627 | 14.36714417 | 16.02691674 | 12.62106219 |
| 0.830757893 | 1.026360461 | 0.96761162 | 1.101268814 | 1.107677191 | 11.74869079 | 12.89881535 | 14.97725587 | 17.17722405 | 19.12995306 |
| 0.960927838 | 1.012230253 | 1.073632315 | 0.978854726 | 1.115381695 | 14.76826146 | 13.82460799 | 13.97427385 | 15.37404617 | 9.433292669 |
| 1.267951885 | 1.335645827 | 1.416666332 | 1.291606554 | 1.47175497 | 19.48683782 | 18.2416796 | 18.4391649 | 20.28617555 | 12.4473043 |

**Figure 7D**

| **Control** | **WT-N** | **R203M/D377Y** | **Delta-N** | **Omicron-N** | **Control+VSV** | **WT-N+VSV** | **R203M/D377Y+VSV** | **Delta-N+VSV** | **Omicron-N+VSV** |
| --- | --- | --- | --- | --- | --- | --- | --- | --- | --- |
| 1.287882 | 0.315344 | 0.989657 | 1.082975 | 0.917004 | 14715 | 114500.9 | 308519 | 293907 | 22149.71 |
| 0.815072 | 0.78187 | 1.010451 | 0.50874 | 0.861546 | 15233.92 | 105362.4 | 445479.6 | 563868.8 | 43088.04 |
| 1.121166078 | 0.552865327 | 1.193335743 | 0.809442217 | 0.955945318 | 13634.74407 | 64858.13863 | 155332.4797 | 591902.4899 | 32429.06932 |
| 0.849685 | 1.010451 | 0.671286 | 0.401925 | 0.256139 | 27459.16 | 25798.51 | 430305 | 332962.5 | 61359.54 |

**Figure S1B**

| Control | 0.0000343 | 0.0000543 | 0.0000643 |
| --- | --- | --- | --- |
| WT-N | 1 | 0.896153 | 1.106975 |
| D63G | 0.934001 | 1.012512 | 1.038786 |
| R203M | 1.005005 | 0.918674 | 1.040663 |
| D377Y | 0.890835 | 0.953081 | 1.019393 |
| D63G/R203M | 1.031279 | 0.984986 | 0.862684 |
| D63G/D377Y | 1.072255 | 0.970597 | 0.913043 |
| R203M/D377Y | 1.001877 | 1.10416 | 0.856741 |
| D63G/R203M/D377Y | 1.095715 | 1.005005 | 0.887707 |

**Figure S1C**

| Control | 0.0000453 | 0.0000643 | 0.0000743 |
| --- | --- | --- | --- |
| WT-N | 1 | 1.100172 | 1.122375 |
| P13L | 1.022031 | 1.010155 | 1.104991 |
| RG203/204KR | 1.02117 | 0.985026 | 1.125473 |
| P13L/RG203/204KR | 1.013425 | 1.089329 | 1.03821 |

**Figure S1D**

| Control | 0.005 | 0.006280249 | 0.005172357 |
| --- | --- | --- | --- |
| WT-N | 1 | 0.846745312 | 1.117287138 |
| D63G | 0.7397 | 1.005819085 | 0.951562836 |
| R203M | 1.1688 | 0.956869339 | 0.983220871 |
| D377Y | 1.0792 | 1.0093 | 1.0435 |
| D63G/R203M | 1.1527 | 1.296873263 | 1.168807628 |
| D63G/D377Y | 1.0189 | 1.049988791 | 0.939766292 |
| R203M/D377Y | 1.0943 | 1.1199 | 1.234 |
| D63G/R203M/D377Y | 1.1368 | 0.927683229 | 0.973968208 |

**Figure S1E**

| Control | 0.0021 | 0.000817402 | 0.001063719 |
| --- | --- | --- | --- |
| WT-N | 1 | 1.023373892 | 1.127660927 |
| P13L | 1.3141 | 1.271619166 | 1.090873765 |
| RG203/204KR | 1.0359 | 1.154018752 | 1.214600635 |
| P13L/RG203/204KR | 0.9794 | 1.231144413 | 1.231144413 |

**Figure S2E**

| Control | 2.176779794 | 2.042976941 | 2.288231335 |
| --- | --- | --- | --- |
| WT-N | 4.694125982 | 4.632654047 | 4.717178023 |
| D63G | 4.759439988 | 4.694125982 | 4.776728998 |
| R203M | 4.784413005 | 4.776728998 | 4.87085798 |
| D377Y | 4.886226007 | 4.909277981 | 4.828595972 |
| D63G/R203M | 4.845885013 | 4.757519011 | 4.697967974 |
| D63G/D377Y | 4.757519011 | 4.730624997 | 4.747914025 |
| R203M/D377Y | 5.485578057 | 5.516314006 | 5.529760955 |
| D63G/R203M/D377Y | 5.735307969 | 5.57202297 | 5.673835988 |
| P13L | 4.865095028 | 4.807465008 | 4.809386029 |
| RG203/204KR | 4.943855997 | 4.941934986 | 4.901593983 |
| P13L/RG203/204KR | 4.30416296 | 4.300321083 | 4.381002926 |

**Figure S2G**

| Control | 2.054586245 | 2.196712157 | 2.060168812 |
| --- | --- | --- | --- |
| WT-N | 5.742991987 | 5.825594999 | 5.733386997 |
| R203M/D377Y | 6.403816037 | 6.520997046 | 6.326975973 |
| D63G/R203M/D377Y | 6.561338034 | 6.597836967 | 6.605520954 |
| RG203/204KR | 6.048430927 | 6.029220988 | 6.056115166 |
| P13L/RG203/204KR | 5.256979062 | 5.28003106 | 5.199348933 |

**Figure S3A**

| Control | 0 | 0 | 0 |
| --- | --- | --- | --- |
| Control | 155 | 142 | 137 |
| WT-N | 86 | 73 | 79 |
| D63G | 81 | 76 | 84 |
| R203M | 72 | 76 | 80 |
| D377Y | 70 | 73 | 61 |
| D63G/R203M | 80 | 73 | 85 |
| D63G/D377Y | 78 | 70 | 83 |
| R203M/D377Y | 51 | 44 | 47 |
| D63G/R203M/D377Y | 45 | 42 | 49 |
| P13L | 84 | 86 | 73 |
| RG203/204KR | 69 | 76 | 68 |
| P13L/RG203/204KR | 86 | 98 | 95 |

**Figure S3B**

|  | **IFN-α** | **IFN-β** | **IFN-γ** |
| --- | --- | --- | --- |
| Control | 1 | 1 | 1 |
| WT-N | 0.856 | 0.6925 | 0.798 |
| D63G | 0.9112 | 0.6759 | 0.9289 |
| R203M | 0.9724 | 0.6399 | 0.9418 |
| D377Y | 0.9418 | 0.662 | 0.8449 |
| D63G/R203M | 0.8928 | 0.687 | 0.7205 |
| D63G/D377Y | 0.951 | 0.6731 | 0.9612 |
| R203M/D377Y | 0.8959 | 0.3767 | 0.6737 |
| D63G/R203M/D377Y | 0.7994 | 0.2119 | 0.727 |
| P13L | 0.9418 | 0.5956 | 0.7835 |
| RG203/204KR | 0.8606 | 0.4792 | 0.7399 |
| P13L/RG203/204KR | 0.9433 | 0.6898 | 0.8982 |

**Figure S3C**

| Mock | 0.506387657 | 0.56652283 | 0.422617571 |
| --- | --- | --- | --- |
| Control | 132.6862114 | 154.5440868 | 235.8743357 |
| WT-N | 139.0078947 | 159.67814 | 106.8188917 |
| D63G | 90.57299101 | 92.47612209 | 102.608682 |
| R203M | 103.6026432 | 124.061959 | 133.8911692 |
| D377Y | 105.5103846 | 86.89740499 | 96.41870778 |
| D63G/D377Y | 122.0714572 | 115.4866355 | 110.0169573 |
| R203M/D377Y | 110.9118071 | 123.0643552 | 124.7822696 |
| D63G/R203M/D377Y | 89.66639332 | 112.7118556 | 108.1204389 |
| P13L | 100.1938033 | 95.44842418 | 100.8907063 |
| RG203/204KR | 124.1732677 | 131.2533842 | 153.9385561 |
| P13L/RG203/204KR | 129.4619469 | 108.8640871 | 131.2691683 |

**Figure S3D**

| Mock | 0.487117035 | 0.452690681 | 0.557544343 |
| --- | --- | --- | --- |
| Control | 101.5530923 | 97.41623763 | 89.02202171 |
| WT-N | 93.70988421 | 64.89922394 | 78.80030571 |
| D63G | 64.98862443 | 64.09390683 | 50.28706007 |
| R203M | 76.16523894 | 73.06258086 | 77.22846606 |
| D377Y | 84.29557525 | 70.88384705 | 80.86172076 |
| D63G/D377Y | 92.58383443 | 86.98461865 | 80.04217188 |
| R203M/D377Y | 71.45428518 | 67.59987289 | 77.65186279 |
| D63G/R203M/D377Y | 104.517577 | 97.51834756 | 103.0786527 |
| P13L | 84.31724276 | 78.12735055 | 75.99095404 |
| RG203/204KR | 105.2237909 | 105.9556802 | 108.1820343 |
| P13L/RG203/204KR | 86.67179064 | 84.30174596 | 85.47855448 |

**Figure S3E**

| Control | 197.3462 | 177.9231 | 196.5385 |
| --- | --- | --- | --- |
| WT-N | 93.83654 | 98.42308 | 93.92308 |
| D63G | 97.38462 | 92.25 | 97.32692 |
| R203M | 91.26923 | 99.375 | 92.45192 |
| D377Y | 93.49038 | 90.98077 | 95.42308 |
| D63G/R203M | 96.43269 | 92.85577 | 96.60577 |
| D63G/D377Y | 88.18269 | 93.20192 | 87.34615 |
| R203M/D377Y | 54.03846 | 52.42308 | 55.90385 |
| D63G/R203M/D377Y | 47.88462 | 51.90385 | 44.21154 |
| P13L | 82.61538 | 95.13462 | 83.94231 |
| RG203/204KR | 83.82692 | 80.30769 | 81.77885 |
| P13L/RG203/204KR | 93.72115 | 89.88462 | 94.70192 |

**Figure S4G**

|  | **RIG-I** | | | **MDA-5** | | |
| --- | --- | --- | --- | --- | --- | --- |
| Control | 252.4280417 | 215.6102962 | 239.9440769 | 267.9135835 | 256.2466081 | 192.3824168 |
| WT-N | 102.1896051 | 135.7120442 | 121.0512202 | 93.27633871 | 87.14285714 | 107.5427922 |
| R203M/D377Y | 48.55730853 | 37.85468428 | 33.94739713 | 94.57275403 | 83.92087995 | 103.7854307 |
| D63G/R203M/D377Y | 36.04510014 | 57.69665154 | 33.00749756 | 113.7049855 | 90.4925471 | 100.5700286 |
| RG203/204KR | 84.13890705 | 72.06918881 | 92.80965123 | 110.0407444 | 90.06765912 | 100.6806057 |
| P13L/RG203/204KR | 148.4346397 | 162.0273939 | 155.984988 | 99.69201505 | 107.7851839 | 116.5986089 |
