# Supplementary material for: The R203M and D377Y mutations of the nucleocapsid protein promote SARS-CoV-2 infectivity by impairing RIG-I-mediated antiviral signaling
Source: PLoS Pathog. 2025 Jan 22;21(1):e1012886. doi: 10.1371/journal.ppat.1012886 (PMC11771877; doi:10.1371/journal.ppat.1012886)
Supplement: Attachment — Submitted filename: Response to Reviewers.docx [file ppat.1012886.s011.docx]

Reviewer's Responses to Questions

**Part I - Summary**
Please use this section to discuss strengths/weaknesses of study, novelty/significance, general execution and scholarship.

Reviewer #1: In this manuscript the authors assessed the impact of two unique mutations that were identified in the N protein of the Delta variant of SARS-CoV-2. During the pandemic, several mutations in N protein have been observed in the different variants. This group used sequence analysis to identify those mutations and then used several genetic techniques to assess the impact of these mutations on both virus replication and interferon production. They compared the impact of these mutations, both singly and in combination, with WT virus as well as with mutations that were identified in the Omicron variant. They concluded that the Delta variant specific mutations promoted SARS-CoV-2 infection and had increased inhibitory activity against RIG-I-dependent IFN production, and thus resulted in reduced levels of IFN following infection with SeV, SARS-CoV-2, or treatment with polyI:C. They then claim that these mutations enhance the affinity of N protein for viral RNA which reduces the ability of RIG-I to interact with viral RNA. Finally, they used AAV to express different N protein mutants in mice and then infect them with VSV, a virus highly sensitive to IFN. They found that the Delta variant N protein and specifically the R203M/D377Y mutant N protein inhibited the production of IFN in mice which led to increased VSV replication and enhanced disease.
Defining the relative importance of variant mutations of SARS-CoV-2 in its ability to replicate and cause disease are critical to understanding how best to target specific variants for treatment and to help identify new variants that may cause increased disease. The authors here have convincingly showed that these two primary N protein mutations in the Delta variant enhance the ability of N protein to promote virus replication, bind RNA, and further enhance N protein’s ability to repress IFN responses. The demonstration that these mutations enhance VSV replication in vivo (for which N protein has no role in replication) strongly demonstrates its IFN inhibitory activity. However, there are several issues with the interpretation that the mechanism of IFN repression is due to reduced RIG-I-RNA interaction. Furthermore, no data was provided that demonstrated that the enhanced SARS-CoV-2 replication is due to reduced IFN production. Thus, the results do not fully support the conclusion stated in the title. Finally, the writing in the paper is not up to the standards of the journal, and thus the paper needs to be extensively edited before it is suitable for publication.

**Authors’ Response:**

Thank you very much for your insightful comments on our paper. In response to your queries, we have provided point-by-point replies below. We have conducted additional experiments using type I interferon blocking antibodies to further demonstrate that the enhanced SARS-CoV-2 replication is due to reduced IFN production (Revised Figure 4G and H). Following your suggestions, we have also carefully refined the language of the paper.

Reviewer #2: Overall, the paper confirms two critical amino acids in the N protein of SARS-CoV-2, where their combination mutant exhibits a dual function: suppressing the RIG-I-mediated interferon production, and enhancing viral replication through increasing RNA binding to the N protein. This discovery sheds light on the mechanisms behind SARS-CoV-2’s variability in viral RNA replication and its capacity to modulate host immune responses.

**Authors’ Response:**

Thank you very much for your comments on our paper.

Reviewer #3: The R203M and D377Y Mutations of N Protein promote SARS-CoV-2 Infectivity through impairing RIG-I-mediated antiviral signaling
PLOS Pathogens
Summary:
The manuscript is discussing the SARS-CoV-2 nucleocapsid mutations that are found in certain variants. These mutations found in the Delta variants are compared to those found in Omicron variants. The authors claim that the N protein mutations found in the Delta variants promoted viral replication. Increase in viral replication caused by N protein mutations in Delta caused inhibition of interaction between RIG-I and MAVS that lead to increased viral replication. Induction of interferons was measured after infection with different interferon activators and to original SARS-CoV-2 virus indicting that the N protein mutant inhibit IFN-Beta but no inhibition of other IFN-types of . Authors investigated the role of the N protein mutations on interacting with other cellular proteins that are involved in activating interferons. N protein with specific mutations inhibited interaction with intracellular RIG-I protein that is activated by binding to viral RNA. Furthermore, the RIG-I and MAVS interaction was inhibited by these N mutations leading to inhibition of phosphorylation of TBK1 and IRF3 proteins. Nucleocapsid mutations phenotypes were also investigated in C57BL/6 mice using AAV system and activation of RIG-I pathway using VSV. The manuscript is well written and investigated SARS-CoV-2 some nucleocapsid mutations with intensive experiments.

**Authors’ Response:**

Thank you very much for your positive comments on our paper.

**Part II – Major Issues: Key Experiments Required for Acceptance**
Please use this section to detail the key new experiments or modifications of existing experiments that should be absolutely required to validate study conclusions.

Generally, there should be no more than 3 such required experiments or major modifications for a "Major Revision" recommendation. If more than 3 experiments are necessary to validate the study conclusions, then you are encouraged to recommend "Reject".

Reviewer #1: 1. The paper has many grammatical errors, incorrect word usage, incorrect statements etc. For example, line 77 indicates that N protein helps form cellular stress granules, when it actually prevents their formation. At multiple places the authors stated that the omicron N protein promoted IFN production compared to the delta strain N protein (i.e. line 190), but in reality, the N protein from omicron represses IFN production, just not to the same extent. The correct terminology is that the omicron N protein had reduced inhibition of IFN production as compared to the delta N protein. Another example is line 163, where it states that the omicron N protein inhibited trVLP replication compared to delta mutant N protein, but again it just promotes virus replication to a lesser degree. The manuscript also lacked references in many places (i.e. line 214) and the title is not well written and has some words have capital letters where there shouldn’t be. The bottom line is that the manuscript requires extensive editing.

**Authors’ Response:**

Thank you very much for your suggestion. According to your suggestion, we carefully revised the grammar, incorrect word usage, incorrect statement and added references in some places in the manuscript. We have also re edited the title of the paper.

2 The major issue in this manuscript is that the data supporting their final model, where the delta N protein has enhanced RNA binding, which results in reduced RIG-I-RNA interactions and reduced IFN production does not hold up when comparing all of the mutants. Most notably, the omicron mutant protein RG203/204KR reduced RIG-I-RNA binding to the same degree as the Delta mutant protein (Fig. 6) but did not have the same reduction in IFN production following SeV, SARS-CoV-2, or polyI:C treatment (Figs. 4, 5). It also had roughly similar levels of RIG-I binding (Fig. 5B) and promoted virus replication to the same degree as the WT protein (Figs 2-3), further indicating that reduced RIG-I-RNA binding does not impact IFN or virus replication.

**Authors’ Response:**

Thank you for the comment. we redraw the model in Figure 8 to show differences among the Original, Delta and Omicron. I understand your concern. When we compared all the mutations, we found that compared to the original N protein, the N mutation of Omicron (P13L plus RG203/204KR) can to some extent inhibit virus replication, but has little effect on interferon, which seems to contradict our previous model. However, our main goal was to demonstrate that the mutations in R203M and D377Y on Delta affect the binding of RIG-I-RNA, thereby inhibiting the RIG-I regulated interferon pathway. Therefore, we have redrawn the model and described them in the Revised manuscript (line 112-116): “It is noteworthy that, compared to the original N protein, mutations at the 13 and 203/204 sites in the Omicron N protein had little effect on interferon expression. However, these mutations inhibited viral replication by suppressing the binding of the N protein to viral RNA.”

As shown in Fig 1D and 1E, the mutations at the two sites, RG203/204KR, first emerged in the Alpha variant. Previous studies have demonstrated that compared to the original strain, these mutations can promote viral replication (Mourier, T., et al., SARS-CoV-2 genomes from Saudi Arabia implicate nucleocapsid mutations in host response and increased viral load. Nat Commun, 2022. 13(1): p. 601. Wu, H., et al., Nucleocapsid mutations R203K/G204R increase the infectivity, fitness, and virulence of SARS-CoV-2. Cell Host Microbe, 2021. 29(12): p. 1788-1801.e6.). The mechanism may involve alterations in the binding of RG203/204KR to its own RNA, interactions with key host proteins, and the assembly of viral RNP. In our experiments, we also demonstrated that compared to the N protein of the original strain, the RG203/204KR mutations enhanced binding to its own RNA and reduced RIG-I-RNA binding ( Revsised Figure 6A-6F). Compared to the Delta variant's mutated N protein (including R203M and D377Y sites), the RG203/204KR mutations decreased binding to its own RNA and increased RIG-I-RNA binding (Revsised Figure 6A-6E) (we had supplemented an analysis of significant differences). In terms of inducing interferon, compared to the Delta variant's mutated N protein (including R203M and D377Y sites), the RG203/204KR mutations somewhat increased interferon expression (Revised Figure 4C, 4E and 4F) (we had supplemented an analysis of significant differences). In Revised Figure 5B, compared to the Delta variant's mutated N protein (including R203M and D377Y sites), the RG203/204KR mutations promoted binding to RIG-I. Compared to the original N protein, the RG203/204KR mutations promoted viral replication to a certain extent (Revised Figure 2C, 2E, 3C, 3E, S2E and 2G). In our model, we aim to illustrate that the R203M and D377Y mutations in the N protein of the Delta variant can reduce RIG-I-RNA binding, thereby regulating the RIG-I-MAVS pathway, inhibiting interferon production, and promoting viral replication. According to your suggestion, we have modified the model diagram to emphasize the role of R203M and D377Y mutations. Although the RG203/204KR mutations indeed enhanced binding to its own RNA and reduced RIG-I-RNA binding, their effects were not as strong as those of R203M and D377Y, only moderately reducing interferon expression and promoting viral replication levels.

Furthermore, the authors never analyzed the impact of N protein on MDA5-mediated induction of IFN, which is important as MDA5 is also a major RNA sensor during CoV infection and poly(I:C) treatment. Though it appears that since the N protein reduced IFN production from SeV and CoV infection, that it probably inhibits IFN production induced from either sensor.

**Authors’ Response:**

Thank you for your good suggestion. According to your suggestion, We had supplemented the experiments regarding the impact of mutated N proteins on MDA5-regulated IFN. Co-immunoprecipitation experiment demonstrated that: the original and mutant N protein could interact with MDA5, and that the mutations in N proteins didn’t alter the interaction with MDA5 compared with original N protein (Revised Figure S4C). The dual luciferase assay for IFN-β show that, compared to the control group, the original N protein significantly inhibited MDA5-induced IFN-β promoter activity. Furthermore, compared to the original N protein, different mutated N proteins do not affect MDA5-induced IFN-β promoter activity (Revised Figure S4G), thereby proving that the differential expression of interferon regulated by these mutated N proteins is not mediated through MDA5.

3. In line with the previous comment, it was odd that the Delta N protein had reduced RIG-I binding. Has it not been shown that the interaction of N protein with RIG-I is critical for its ability to repress IFN production? Or is this unknown? Perhaps they only interact through their ability to bind RNA, which might help explain the data. The authors should perform Co-IPs with N protein and RIG-I in the presence of RNase (or at least in the absence of RNA) to determine if the interaction between the two proteins is dependent on RNA binding. They have recombinant proteins and could easily do this in vitro. Others have shown that N protein interacts with TRIM25 which reduces the ubiquitination of RIG-I. It would be prudent to also determine if the Delta N protein impacts RIG-I-TRIM25 interactions and its ubquitination. (PMIDs:33801464; 32295922; 28148787)

**Authors’ Response:**

Thank you for your comments and suggestion. The interaction between the N protein of the original SARS-CoV-2 strain and RIG-I, which inhibited the production of interferon, had been demonstrated in a previously published article by our lab (Chen, Keli et al. “SARS-CoV-2 Nucleocapsid Protein Interacts with RIG-I and Represses RIG-Mediated IFN-β Production.” *Viruses* vol. 13,1 47. 30 Dec. 2020,). To further investigate the relationship between the N-RIG-I interaction and RNA, according to your suggestion, we conducted an in vitro pull-down experiment using purified N mutant proteins and RIG-I protein. The results showed that, compared to the control group, there is a direct interaction between the original N protein and RIG-I protein. Compared to the original N protein, the N protein of the Delta variant (containing R203M and D377Y mutations) significantly inhibited its interaction with RIG-I. In contrast, compared to the Delta variant N protein, the N protein of the Omicron variant promoted its interaction with RIG-I (Revised Figure 5B). These results are consistent with the co-immunoprecipitation (co-IP) results obtained in cells (Revised Figure 5A and S4D), indicating that there was a direct interaction between N and RIG-I, and this interaction was not affected by RNA binding.

Other studies have found that the N protein can interact with TRIM25, thereby reducing TRIM25-mediated ubiquitination of RIG-I (Gori Savellini, Gianni et al. “SARS-CoV-2 N Protein Targets TRIM25-Mediated RIG-I Activation to Suppress Innate Immunity.” Viruses vol. 13,8 1439. 23 Jul. 2021,). We first investigated the impact of different mutated N proteins on the interaction between TRIM25 and RIG-I. Co-IP results demonstrated that, compared to the control group, the original N protein significantly inhibits the interaction between TRIM25 and RIG-I. Compared to the original N protein, different mutated N proteins had no effect on the interaction between TRIM25 and RIG-I (Revised Figure S6A). Ubiquitination experiments of RIG-I showed that, compared to the control group, the original N protein significantly inhibited TRIM25-mediated ubiquitination of RIG-I . Compared to the original N protein, different mutated N proteins do not affect TRIM25-mediated ubiquitination of RIG-I (Revised Figure S6B).

4. There was no data to support the conclusion that “R203M and D377Y Mutations of N Protein promote SARS-CoV-2 Infectivity through impairing RIG-I-mediated antiviral signaling”. The authors did not perform any experiment that specifically demonstrated that IFN signaling impacted virus replication in these cells. This would be required to make this conclusion.

**Authors’ Response:**

Thank you for your good question. To further demonstrate that the mutated N protein promoted its own replication by inhibiting the production of interferon, we first transfected Caco-2 cells with different mutated N proteins, and then added Anifrolumab, a type I interferon receptor antagonist, which can block the activity of type I interferons (Kretzschmar, Genia et al. “Normalized Interferon Signatures and Clinical Improvements by IFNAR1 Blocking Antibody (Anifrolumab) in Patients with Type I Interferonopathies.” Journal of clinical immunology vol. 45,1 31. 23 Oct. 2024). Subsequently, the cells were infected with VLP (Revised Figure 4G) and SARS-CoV-2 live virus (Revised Figure 4H), respectively. The results showed that, Delta N protein having mutations containing sites R203M and D377Y promoted SARS-CoV-2 replication more strongly than the Omicron mutant N protein (containing sites P13L and RG203/204KR) and original N protein; the mutations P13L and RG203/204KR in the Omicron mutant N protein reduced its activity on promoting viral replication compared with original N protein. However, after pre-treated with Anifrolumab, the different mutated N proteins had no effect on the replication of VLP (Revised Figure 4G) and SARS-CoV-2 live virus (Revised Figure 4H).

Reviewer #2: Generally, there should be no more than 3 such required experiments or major modifications for a "Major Revision" recommendation. If more than 3 experiments are necessary to validate the study conclusions, then you are encouraged to recommend "Reject".
Three new experiments are needed: 1) compare the viral growth kinetic of the original strain, the Delta and the Omicron variants in Calu-3 cells or Caco-2 cells. After infection, at various time points post-infection (eg. 6, 12, 24, 48 and 72 hours) collect supernatants and measure viral titers to quantify infectious particles. At these time points, the authors should detect the type I interferon production by qPCR or ELISA to assess the differences in IFN production induced by these three strains;

**Authors’ Response:**

Thank you for your good suggestion. According to your suggestion, We compared the viral growth kinetic and interferon changes of the original strain, Delta variant, and Omicron variant in Caco-2 cells at various time points post-infection (eg. 6, 12, 24, 48 and 72 hours). The experimental results showed that compared to the original strain, the Delta variant exhibited significantly enhanced replication capability starting from 12 hours post-infection, which corresponded to a significant decrease in the level of interferon produced by the infected cells (Revised Figure 4A and 4B). In contrast, compared to both the original strain and the Delta variant, the Omicron variant demonstrated a significant reduction in replication capability starting from 6 hours post-infection, accompanied by a significant increase in the level of interferon produced by the infected cells (Revised Figure 4A and 4B). These findings suggest that the differences in replication dynamics among the three different variants may be associated with their ability to induce interferon.

2) In figure 5 E, a notable observation is that IRF3 was not detected in all cells especially in control group. The experiment needs further investigation.

**Authors’ Response:**

Thank you for your question. Due to the use of endogenous IRF3 antibody, which may be influenced by antibody specificity, IRF3 may not be detectable in every cell; additionally, uneven cell distribution and cell attachment could also contribute to the inability to detect IRF3 in every cell. We enhanced the brightness of IRF3 (Revised Figure 5E) to ensure that IRF3 was detected in most cells.

Reviewer #3: (No Response)

**Part III – Minor Issues: Editorial and Data Presentation Modifications**
Please use this section for editorial suggestions as well as relatively minor modifications of existing data that would enhance clarity.

Reviewer #1: (No Response)

Reviewer #2: Minor modifications: 1) SARS-CoV-2 has a complex genome that includes several open reading frames (ORFs) with key roles in viral pathogenesis. Notably, ORF3, ORF6, and ORF9b are critical for antagonizing the host's innate immune response. These ORFs interfere with the host’s antiviral defenses by disrupting interferon signaling and other immune mechanisms, thereby facilitating viral persistence and replication. Among these, ORF6 has been identified as crucial for viral replication. Particularly noteworthy is the D61L mutation in ORF6, which emerged in the Omicron strains. This mutation impairs immune evasion, highlighting its essential role in the virus’s ability to propagate. Please update introduction section with these specific details;

**Authors’ Response:**

Thank you for your good suggestion. According to your suggestion, We have added relevant descriptions in the introduction. As follows: SARS-CoV-2 has a complex genome that also includes several open reading frames (ORFs) with key roles in viral pathogenesis. Notably, ORF3[19], ORF6[20] and ORF9b[21] are critical for antagonizing the host's innate immune response. These ORFs interfere with the host’s antiviral defenses by disrupting interferon signaling and other immune mechanisms, thereby facilitating viral persistence and replication. Among these, ORF6 has been identified as crucial for viral replication. Particularly noteworthy is the D61L mutation in ORF6, which emerged in the Omicron strains. This mutation impairs immune evasion, highlighting its essential role in the virus’s ability to propagate.

3) The structure of various N protein variants is illustrated in figure 1B. This figure only presents the mutant sites of N protein but does not provide a detailed description of the differences between these variant structures;

**Authors’ Response:**

Thank you for your question. According to your suggestion, We have added relevant descriptions in the manuscript. As follows: The structures of those two mutant proteins were relatively conserved, with D63G located in the NTD, R203M located in the linkage region and D377Y located in the C-terminus of the Delta mutant N protein, while Omicron variant including P13L located in the N-terminus and R203K, G204R mutation located in linkage region

4) The legends for figure 2B and 2D are currently unclear and do not provide a detailed description. The figure legends for figure 2B and 2D, as well as for Figure 2C and 2E, are identical and do not clearly describe the differences between the two experiments;

**Authors’ Response:**

Thank you for your question. According to your suggestion, We have added relevant descriptions in the manuscript. As follows: (**B-C**) Caco-2 cells were transfected with plasmids encoding different mutated N protein for 24 h, and then infected with SARS-CoV-2 GFP/ΔN (MOI=0.5) for 2 h, washed and incubated for an additional 48h. GFP expression was observed in Caco-2 cells using microscopy at indicated time after inoculation (B). (**C**) The total RNA in supernatant of Caco-2 cells was extracted and RT- qPCR assays were conducted to determine viral copies. Cell lysates were analyzed by immunoblotting. (**D-E**) The above cell culture medium were collected to infect the Caco-2-N cells for 48h. GFP expression was observed in Caco-2-N cells using microscopy after inoculation for 48h (D). (**E**) The total RNA in supernatant of Caco-2-N cells was extracted and RT- qPCR assays were conducted to determine viral copies.

5) In figure 4, it was observed that mutant of N protein inhibits the production of interferon-beta but does not affect interferon-alpha production. Please discuss the reasons for this selective inhibition in Discussion section;

**Authors’ Response:**

Thank you for your suggestion. According to your suggestion, we have added relevant descriptions in the discussion section. As follows: We found that the mutant N protein inhibited the production of IFN-β, but has no effect on the production of IFN-α. We speculated that this may be due to two reasons. On one hand, in the cells we used, the expression of IFN-β is higher than that of IFN-α, making it more susceptible to changes and detection. On the other hand, there are multiple homologous genes for IFN-α in the genome, and our primers may not be able to accurately assess the overall expression of IFN-α.

6) The manuscript contains many awkward/incorrect uses of words and phrases. The authors need to use a science editing service that employs native English speakers, as these problems make the manuscript difficult to read.

**Authors’ Response:**

Thank you for your suggestion. According to your suggestion, we have also carefully refined the language of the paper.

Reviewer #3: Here are some notes to the authors to address before resubmitting the manuscript.
Comments:
1. There are a couple of studies about SARS-CoV-2 detect a role of IFN type III. Could you elaborate of not measuring type III IFN beside the other interferon in your study?

**Authors’ Response:**

Thank you for your good question. In fact, we did detect type III interferons in our experiments, but the expression levels of type III interferons were very low in the cells we used, including Caco-2 and HEK293T, and there were no significant differences observed. Therefore, we did not present those results.

2. I would like to know what strain you refer to by original SARS-CoV-2 strain, and its accession number. (e.g. Wuhan strain)

**Authors’ Response:**

Thank you for your good question. The original SARS-CoV-2 strain (accession number is MN908947.3)

3. Figure 2A, the first part of “de novo infection” of VLP is not accurate as stated in the protocol and in the text. Needs more clarifications or modifications to fit with the procedure in the protocol.

**Authors’ Response:**

Thank you for your good question. We changed “de novo infection” to“ First infection”in Figure 2A, 3A S2C.

4. Fig 2D, it would be better to write the time of infection in the figure.

**Authors’ Response:**

Thank you for your good suggestion. We added the time of infection in the Figure 2D

5. Figure 3A, the first part of “de novo infection” of SARS-CoV-2 is not accurate as stated in the protocol and in the text. Infection of original SARS-CoV-2 was after transfecting the cells with N and N-mutant. This needs more clarifications or modifications to fit with the procedure in the protocol.

**Authors’ Response:**

Thank you for your good suggestion. we have added relevant descriptions in the Figure 3A legends. As follows: Experimental scheme. Caco-2 cells were firstly transfected with plasmids encoding different mutated N protein for 24 h, and then infected with SARS-CoV-2 (MOI=0.5) for 2 h, washed virus and incubated fresh maintenance culture medium for an additional 24h. Viral RNA in cell and viral copies in supernatant was determined by RT-qPCR assay. The cell culture medium include virus was collected to infect the untransfected Caco-2 cells for another 24h. Viral RNA in cell and viral copies in supernatant was determined by RT-qPCR assay.

6. I would suggest that the viral copies per mL to be graphed as Log scale to visual the difference between mutants easily.

**Authors’ Response:**

Thank you for your good suggestion. According to your suggestion, we changed “viral copies per mL” to“ viral copies log_10_ mL”in Revised Figure 2C, 2E, 3C, 3E, 4G, 4H, S2E, S2G.

7. Line 171, “The SARS-CoV-2 in supernatant continued to infect Caco-2 cells…” that is a second infection?!

**Authors’ Response:**

Thank you for your question. “The SARS-CoV-2 in supernatant continued to infect Caco-2 cells…” is a second infection.

8. Line 186, “Sev” I assume you refer to Sendai virus, you should state that before the acronym.

**Authors’ Response:**

Thank you for your question. We had added Sendai virus (Sev) in the revised manuscript.

9. I would suggest that the SARS-CoV-2-trVLP work should be sectioned in 2 parts in the text. The SARS-CoV-2-trVLP work on Caco-2 cells should be separated from the HEK293T-ACE2 cells as these cells have been used as a confirmatory data and placed in the supplementary data section. (Section start from line 137 to 165)

**Authors’ Response:**

Thank you for your suggestion. According to your request, the SARS-CoV-2-trVLP work was sectioned in 2 parts in the text.

10. Line 224, “co-location” should be “co-localization”

**Authors’ Response:**

Thank you for your suggestion. We had changed “co-location” to “co-localization” in the revised manuscript

11. Fig 6E right, it would be better to label the proteins as in the other figures. I would probably move this figure to the supplementary file.

**Authors’ Response:**

Thank you for your suggestion. According to your suggestion, we moved Original Figure 6E to the Revised supplementary Figure S4E and 4F and labeled the proteins as in the other figures

12. Line 287, “(incubation with the same quality of RIG-I protein and viral RNA …), you mean quantity (ug) not quality.!?

**Authors’ Response:**

Sorry for using the wrong word, it was incubation with the same quantity of RIG-I protein and viral RNA

13. Since the phenotype of Omicron variant N mutation at P13L behave different when combined with R203G/K204R, can you elaborate on the function of the P13L mutation in Omicron variant.

**Authors’ Response:**

Good question. We hypothesize that compared with the R203G/K204R, combined P13L with R203G/K204R mutations may alter the binding of N protein to self RNA and RIG-I-RNA, as demonstrated in Figures 6A-F.

14. Figure 8 that summarizes the phenotype of Delta and Omicron nucleocapsid variants is not clear enough, could you please redraw that figure with more clarification indicating what is inhibited and what is increased to show differences between the 2 variants.

**Authors’ Response:**

Thank you for your suggestion. According to your suggestion, we redraw the model in Figure 8 to show differences between the 2 variants.

15. I know how difficult it is to make infectious clones with specific mutations in the nucleocapsid, but the conclusion of the results should exclude that the phenotype observed in this manuscript using trVLP system would be similar to the phenotype of mutations using actual infectious SARS-CoV-2 clones harbouring the same mutations. These comments should be stated very clearly in the discussion, especially for interpreting mice data.

**Authors’ Response:**

Thank you for your professional cognition, understanding and good suggestion. According to your suggestion, we have added relevant descriptions in the discussion. As follows: Notably, the phenotypic effects of these mutations were observed using the trVLP system. Ideally, these findings should be confirmed using actual infectious SARS-CoV-2 clones harboring the same mutations, but technically it is difficult to make infectious clones with specific mutations in the nucleocapsid.
