# Supplementary material for: The R203M and D377Y mutations of the nucleocapsid protein promote SARS-CoV-2 infectivity by impairing RIG-I-mediated antiviral signaling
Source: PLoS Pathog. 2025 Jan 22;21(1):e1012886. doi: 10.1371/journal.ppat.1012886 (PMC11771877; doi:10.1371/journal.ppat.1012886)
Supplement: Attachment — Submitted filename: Response_to_Reviewers_auresp_2.docx [file ppat.1012886.s012.docx]

Reviewer's Responses to Questions

**Part I - Summary**
Please use this section to discuss strengths/weaknesses of study, novelty/significance, general execution and scholarship.

Reviewer #1: The authors have addressed all of my concerns, having done some really nice new experiments to better prove their overall conclusions.
There are just a couple of minor grammatical changes yet to be made.
Line 31. This should be "Viral protein mutations......"
Line 294. "inhibited the direct interaction"
Line 296. Should be Fig. 5B
Line 307. "both N protein mutations R203M and D377Y"
Line 380. The first sentence of this paragraph is incomplete.

**Authors’ Response:**

Thank you very much for your positive comments on our paper. According to your suggestion, we carefully revised the grammar which your mentioned in the revised manuscript.

Reviewer #2: In this manuscript, the authors investigated the functional impact of two key amino acids mutations in the N protein of the SARS-CoV-2 Delta variant. Compare to omicron variant, the N protein mutations in the Delta variant enhanced viral replication. Mechanistic studies revealed that the N mutants bound to RIG-I, suppressing interferon production and evading the host immune response. Furthermore, the N protein mutants exhibited enhanced RNA-binding capability, directly promoting viral replication. These findings illuminate the mechanism underlying SARS-CoV-2’s variability in viral RNA replication and its ability to modulate host immune responses.

**Authors’ Response:**

Thank you very much for your positive comments on our paper.

Reviewer #3: The modification made to this manuscript is sufficient for publication

**Authors’ Response:**

Thank you very much for your positive comments on our paper.

**Part II – Major Issues: Key Experiments Required for Acceptance**
Please use this section to detail the key new experiments or modifications of existing experiments that should be absolutely required to validate study conclusions.

Generally, there should be no more than 3 such required experiments or major modifications for a "Major Revision" recommendation. If more than 3 experiments are necessary to validate the study conclusions, then you are encouraged to recommend "Reject".

Reviewer #1: (No Response)

Reviewer #2: I have read the response to the reviewer's comments and I am satisfied that the authors have made reasonable efforts to address these comments. A far as I am concerned they have answered all my queries and I think the manuscript is much improved.

**Authors’ Response:**

Thank you very much for your positive comments on our paper.

Reviewer #3: (No Response)

**Part III – Minor Issues: Editorial and Data Presentation Modifications**
Please use this section for editorial suggestions as well as relatively minor modifications of existing data that would enhance clarity.

Reviewer #1: (No Response)

Reviewer #2: All of my original comments have been addressed.

**Authors’ Response:**

Thank you very much for your positive comments on our paper.

Reviewer #3: (No Response)
